# Novel Biomarkers for Diagnosis and Monitoring of Immune Thrombocytopenia

**DOI:** 10.3390/ijms24054438

**Published:** 2023-02-23

**Authors:** Alessandro Allegra, Nicola Cicero, Giuseppe Mirabile, Concetto Mario Giorgianni, Sebastiano Gangemi

**Affiliations:** 1 Division of Hematology, Department of Human Pathology in Adulthood and Childhood “Gaetano Barresi”, University of Messina, 98100 Messina, Italy; 2 Department of Biomedical, Dental, Morphological and Functional Imaging Sciences (BIOMORF), University of Messina, 98100 Messina, Italy; 3 Allergy and Clinical Immunology Unit, Department of Clinical and Experimental Medicine, University of Messina, 98100 Messina, Italy

**Keywords:** immune thrombocytopenia, platelet, biomarker, immune system, autoimmune disease, oxidative stress, diagnosis, prognosis, response to treatment

## Abstract

Lower-than-normal platelet counts are a hallmark of the acquired autoimmune illness known as immune thrombocytopenia, which can affect both adults and children. Immune thrombocytopenia patients’ care has evolved significantly in recent years, but the disease’s diagnosis has not, and it is still only clinically achievable with the elimination of other causes of thrombocytopenia. The lack of a valid biomarker or gold-standard diagnostic test, despite ongoing efforts to find one, adds to the high rate of disease misdiagnosis. However, in recent years, several studies have helped to elucidate a number of features of the disease’s etiology, highlighting how the platelet loss is not only caused by an increase in peripheral platelet destruction but also involves a number of humoral and cellular immune system effectors. This made it possible to identify the role of immune-activating substances such cytokines and chemokines, complement, non-coding genetic material, the microbiome, and gene mutations. Furthermore, platelet and megakaryocyte immaturity indices have been emphasized as new disease markers, and prognostic signs and responses to particular types of therapy have been suggested. Our review’s goal was to compile information from the literature on novel immune thrombocytopenia biomarkers, markers that will help us improve the management of these patients.

## 1. Introduction

### General Considerations for Immune Thrombocytopenia

Adults and adolescents can develop immune thrombocytopenia (ITP), an acquired immune-mediated disease. ITP is defined by a temporary or permanent decrease in platelet count and, depending on the degree of thrombocytopenia, by an elevated risk of bleeding [1]. Due to its chronic nature, ITP has a substantially higher prevalence and has a global incidence of 1–5/100,000 [2].

**Figure 1:**
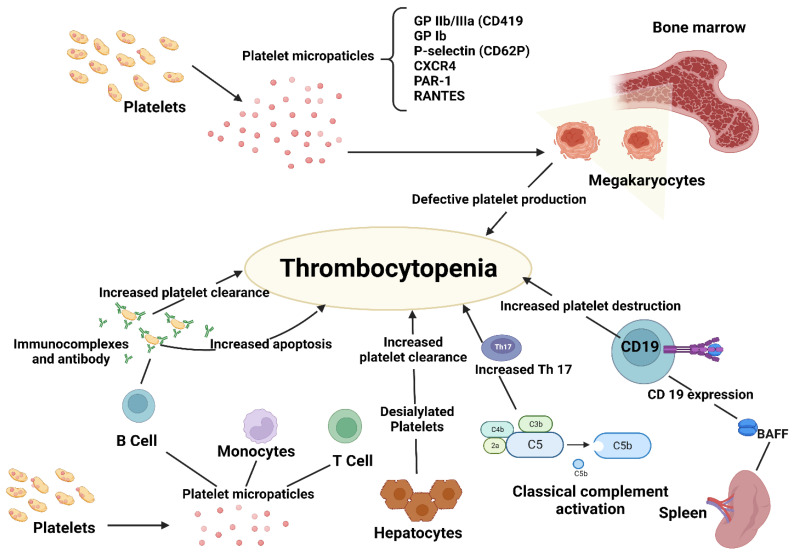
Pathogenesis of immune thrombocytopenia.

The predominant pathogenetic theory, which is also supported by the test-therapeutic effects of therapies such as immunoglobulins and splenectomy, has been that antibodies cause platelet destruction. This theory about the pathophysiology of thrombocytopenia, however, has changed from an emphasis on increased platelet destruction caused by autoantibodies to more complicated processes where both decreased platelet synthesis and T-cell-mediated effects are involved [3,4] (Figure 1).

In reality, the formation of autoreactive cytotoxic T lymphocytes against megakaryocytes, which impairs megakaryopoiesis, represents a distinct mechanism for ITP. Initiating events include Helicobacter pylori infections with an ambiguous mechanism and viral infections that exhibit a type of molecular mimicry. ITP can also develop as a result of immunological dysregulation in lymphomas and other systemic autoimmune disorders [5].

Over two-thirds of adult patients who receive current treatments for chronic ITP, including corticosteroids, thrombopoietin receptor agonists, rituximab, and splenectomy, see an improvement in their platelet count to a stable level. However, a deeper understanding of the pathogenesis of ITP and an earlier, more accurate diagnosis are urgently needed because some patients continue to respond insufficiently to present treatments [6].

In actuality, the emergence of autoreactive cytotoxic T cells represents a distinct mechanism for ITP. Since there is no particular biomarker at this time, the diagnosis of ITP is still an exclusion [7]. Current recommendations state that ITP can be identified in individuals with isolated thrombocytopenia, a platelet count of 100 × 10^9^ L, anaemia or leukopenia, and no other thrombocytopenia-causing conditions [8]. In practical practice, the best proof that a patient has ITP is when he responds to therapy designed specifically for that condition.

The lack of a trustworthy biomarker or gold-standard diagnostic test, despite the ongoing search for one, adds to the high likelihood of disease misdiagnosis. This review summarizes the state-of-the-art research in identifying novel biomarkers that can aid in ITP diagnosis, forecast prognosis, and direct therapeutic treatment through indices that can assess patient receptivity to potential therapies.

## 2. Biomarkers and ITP Diagnosis

### 2.1. Detection of Platelet Autoantibodies and Analysis of Platelet Antigens

Beginning with an understanding of the pathophysiology of the disease, the initial indicators utilized for ITP diagnosis were found. Given that ITP is an autoimmune disease, it was only natural to check for the presence of certain autoantibodies or research antigenic changes on the surface of platelets that could trigger an unwarranted immune response.

By using the modified monoclonal antibody immobilization of platelet antigen (MAIPA) assay, it is possible to identify glycoprotein (GP) specific autoantibodies, such as GPVI, GPIb/IX, and GPIIb/IIIa autoantibodies, in the majority of platelet or plasma eluates from ITP patients. These antibodies bind to the targeted glycoproteins via an antigen-binding fragment (Fab), which then activates the mononuclear-macrophage or complement system [9,10].

About 75% of platelet autoantigens are concentrated in the GP IIb/IIIa or GP Ib/IX complex of the platelet. The antigenic repertoire in chronic ITP may be constrained, as evidenced by the inhibition of the binding of autoantibodies from several ITP patients by either another ITP autoantibody or a monoclonal anti-GPIIb/IIIa antibody. Multiple antibodies may be produced by many patients [11].

The accurate detection of platelet autoantibodies would support the clinical diagnosis, but their utility in the thrombocytopenia diagnostic workup is constrained by the low specificity and sensitivity of the currently available methods for platelet autoantibody testing. The development of techniques for glycoprotein-specific autoantibody detection has increased test specificity and made it acceptable to diagnose ITP but not necessarily exclude it.

Even within studies utilizing assays that are similar, the sensitivity of these tests differs greatly. It is evident from numerous studies that this variation can be accounted for by variations in the test characteristics, such as variations in the glycoprotein-specific monoclonal antibodies, the glycoproteins that are tested, the platelet numbers used in the assay, and the cut-off levels for positive and negative results, as well as differences in the patient populations that were subjected to the tests. It may be able to further standardize and optimize direct autoantibody detection techniques to boost sensitivity without sacrificing specificity, but this will probably not be enough to separate the background signals from the frequently very weak specific autoantibody signals [12]. Therefore, additional advancements in autoantibody detection technologies will be required to boost sensitivity to a level suitable for ITP diagnosis.

In ITP, there was a reduction in the expression of FC gamma receptors (FCGR) IIb on macrophages. The pathophysiology of ITP may be influenced by lower expression of FCGRIIb. A novel biomarker for ITP analysis may be the variation in FCGRIIb [13].

As a final point, serologic analyses for anti-nuclear antibodies (ANAs) play relevant roles in the identification of systemic rheumatic diseases. About 25–39% of ITP subjects have measurable ANAs [14], although their clinical significance is not clear. It has been reported that the positivity of ANAs in ITP subjects was associated with a more chronic course and a greater risk of developing systemic autoimmune disorders [15].

In a study, TP subjects with a positive ANA test were likely to attain a better early response to rituximab administration, while their long-term outcome was adverse. Thus, an ANA test could be useful for predicting rituximab response in ITP [16]. Similarly, ANA positivity in ITP may indicate unresponsiveness to eltrombopag treatment [17].

### 2.2. Immature Platelet Fraction and Megakaryocyte Maturation Index as Diagnostic Biomarkers

An increasing body of research outlines potential biomarkers that could support an ITP diagnosis. The immature platelet fraction (IPF) is one of these markers, which is easily accessible in contemporary automated haematology analysers with fluorescence capacity.

Young platelets, also known as reticulated platelets, that have just been recently liberated from the bone marrow by megakaryocytes, constitute a particular population of platelets. It has been postulated that these platelets are more reactive than mature platelets because they contain modest amounts of RNA within the cytoplasm [18]. IPF determination is a low-cost, dependable, and reliable method for reticulated platelet evaluation that has made it possible to screen for and distinguish between thrombocytopenia from various causes [19].

The IPF (%) value was multiplied by the platelet count to obtain the absolute immature platelet count (AIPC) value, which represents the absolute number of reticulated platelets [20]. IPF can be precisely detected in blood samples even 24 h after they have been drawn [21], most likely because they live longer than mature platelets. Furthermore, by examining immature platelets, consumptive thrombocytopenic processes and those defined by platelet hypoproduction can be easily distinguished [22]. Thus, these counts may indicate whether the cause of the thrombocytopenia is central (originating in the bone marrow) or peripheral (originating elsewhere) [23]. The production of immature platelets does not appear to be impacted by gender [24] or age, as it continues even in older people with decreased platelet counts [25]. Different medications, however, can have an impact on IPF, and when immune responses target platelets, their levels can rise significantly over reference ranges [26,27].

According to reports, bone marrow attempts to counteract platelet destruction by significantly increasing the %-IPF to deal with the consumptive/destructive process [28,29,30]. These increases appear to be greater in people with chronic ITP [31], and these dynamics may help stratify patients at risk of bleeding as they seem to have a higher IPF level [32,33]. Immature platelets are therefore comparable to reticulocyte counts in the presence of anaemia and offer the clinician important information for treating thrombocytopenic patients. The closest thing to real-time information about the bone marrow’s reaction to the aetiology producing the thrombocytopenia is provided by these immature platelet counts [34].

In comparison to a control group, patients with ITP had a higher IPF value and a lower platelet count, according to a study. In an experiment, the IPF values were 13.80% and 3.00%, respectively, for the ITP and control group. The area under the curve for the IPF cut-off value with the highest sensitivity and specificity was 0.973, and the cut-off value was 6.3% [35].

Other studies have confirmed the possibility to use IPF to distinguish thrombocytopenia for platelet consumption, supporting its utility in research into the causes of thrombocytopenia [36]. In their investigation, Serramando et al. found that an IPF cut-off of 11.7% had a sensitivity of 88.2% and a specificity of 91.5% [37]. Their results show that IPF can assess platelet recovery in patients with thrombocytopenia and suggest that IPF has the discriminatory capability to identify the causes of thrombocytopenia.

It has also been discovered that abnormal indicators of ITP megakaryocyte maturation exist. The protein biomarker known as tumour necrosis factor-related apoptosis-inducing ligand (TRAIL) is a member of the TNF superfamily. Megakaryocyte maturation and apoptosis can both be aided by TRAIL. It was shown that reduced platelet formation in ITP was caused by low expression of TRAIL in megakaryocytes. Megakaryocyte TRAIL expression was downregulated, as were patient TRAIL concentrations. A proposed mechanism by which the megakaryocyte number increases in vitro may be the megakaryocyte death caused by TRAIL in the plasma of ITP patients [38]. An alteration of megakaryocyte maturation indices could be a useful parameter for the evaluation of bone marrow replicative dynamics.

### 2.3. Chemokines and ITP Diagnosis

Some chemokines also appear to encompass a fundamentally similar meaning in terms of cellular maturity. Chemokines are tiny proteins that attach to receptors on different leukocyte types to regulate chemotactic activity and the movement of cells. Chemokines are classified into the C, CC, CXC, and CX3C families based on the conserved cysteine motif, with the CC and CXC chemokines receiving the most attention [39]. Several members of the CXC family, such as CXCL12 and its ligand (CXCR4), contribute to migration, homing, proliferation, and survival of hematopoietic stem cells [40]. Wang et al. investigated the megakaryocyte lineage from CFUMeg to platelets and found that CXCR4 was expressed in these cells [41]. In their research, flow cytometry analysis of this receptor’s expression revealed that CXCR4 expression increases with maturation and becomes virtually uniform in the final stages in circulating platelets, exhibiting the greatest expression level in circulating platelets.

In a study, real-time PCR was used to examine CXCR4 gene expression in ITP patients both before and after treatment. Commonly, corticosteroids (prednisone, prednisolone, dexamethasone, or methylprednisolone) or immunoglobulins (IVG) are used as the first-line treatment. All of the patients in this study were new cases; therefore, they all received first-line treatment for a duration of 5–7 days. The expression of the CXCR4 gene showed a significant decrease in comparison with the control group, while its expression did not change before or after treatment [42].

However, the CXCR4 level is likely different in acute and chronic ITP and also in different stages of disease progression. Moreover, several studies have examined the role of this chemokine receptor in various diseases, including systemic lupus erythematosus, HIV and hematologic malignancies such as acute myeloid leukemia, acute lymphoid leukemia, essential thrombocythemia and aplastic anemia. Although CXCR4 is likely to be a mediator with several cellular functions in different stages of maturity of platelets and megakaryocytes, the use of this biomarker must take into consideration these data and the possibility that an alteration of the chemokine expression may be attributable to other causes.

However, a different meaning could be the analysis of a different chemokine. A tiny cytokine from the CXC chemokine family, CXC chemokine ligand-13 (CXCL13), is mostly released by secondary lymphoid tissue, lymph glands, and serum follicular dendritic cells [43]. B1 cell homing, the synthesis of natural antibodies, and body cavity immunity all depend on CXCL13 [44]. Additionally, it has been noted that CXCL13 is a therapeutic target for a number of immunological illnesses and it is essential for the recruitment of B cells and T-cell subsets in pathological situations [45].

ITP patients had higher levels of CXCL13, according to research [46]. Children with ITP reported increased plasma CXCL13 compared to controls; however, this concentration decreased after treatment. Dexamethasone reduced CXCL13 levels in vitro in a dose- and time-dependent manner. As for the mechanism, it was demonstrated that in CD4+ T cells, miR-125-5p mimics lowered the CXCL13 level, whereas an miR-125-5p inhibitor boosted the CXCL13 level. MiR-125-5p was suggested to have CXCL13 as a target gene. A reduction in CXCL13 caused by dexamethasone was likewise prevented by the miR-125-5p inhibitor. The miR-125-5p target gene, CXCL13, may play a role in the pathogenesis of ITP and also serve as a disease marker [47].

Finally, immune problems and significant heterogeneity were present in ITP patients with CCR7, and it was shown that CCR7 was implicated in the disease’s development. In comparison to healthy controls, pretreatment ITP patients had higher CD4/CD8 ratios, lower levels of NK cells and CD4+CD25+CD127low Tregs, and lower levels of NK cells. In comparison to the relapsed group, the newly diagnosed group showed a greater CD4/CD8 ratio and more NK cells. Treg levels were higher in the group experiencing remission than in the group experiencing recurrence. When compared to controls and the remission group, the newly diagnosed and relapsed groups showed larger increases in the CD4+CCR7+, CD8+CCR7+, and CCR7+ subsets of B cells and NK cells. In comparison to the newly diagnosed group, the values for the CD4+CCR7+ and CD8+CCR7+ subsets in the relapsed group were marginally higher. When compared to the relapsed group, the CCR7+ subsets of CD4+ T-cells, CD8+ T-cells, NK cells, and B cells in the remission group had lower levels. The remission group had higher levels of the CD8+CCR7+ subset and lower levels of NK cells than the controls. ITP patients had a lower ratio of the CD4+CCR7+ to CD8+CCR7+ subsets than did healthy controls. The CD8+CCR7+ subgroup and platelet count in ITP patients had a negative correlation [48]. 

### 2.4. BAFF and Autoreactive Cells

As our understanding of the aetiology of ITP has increased, other diagnostic indicators have been identified. The family of tumour necrosis factor ligands member B-cell activating factor (BAFF), also known as B lymphocyte stimulator, is essential for maintaining proper B-cell growth, homeostasis, autoreactivity, and T-cell costimulation [49,50].

It has been demonstrated that BAFF promotes CD19 expression and mediates the development of autoreactive B cells [51,52,53]. It has been reported that high BAFF can prevent the death of autoreactive B and T cells [54]. Compared to patients in remission and controls, individuals with active illness showed greater levels of plasma BAFF and BAFF mRNA. In in vitro tests, rhBAFF promoted the survival of of CD8+ and CD19+ cells. These results imply that BAFF may contribute to the pathogenesis of ITP by enhancing CD19+ and CD8+ cell survival, and enhancing platelet death.

The importance of BAFF expression in pediatric ITP patients was recently assessed by a study. Three groups of pediatric ITP patients have been selected. Group I contained patients with acute ITP, group II patients with persistent ITP, and group III formed by healthy controls. Compared to controls, BAFF expression levels considerably increased in ITP patients. Groups I and II, however, had equivalent BAFF expression values [55]. These findings support BAFF’s potential involvement in the illness and its inclusion in the diagnostic constellation.

### 2.5. Immune Tolerance and ITP: The Role of Interleukins in Secondary Thrombocytopenias

The fundamental cause of ITP’s onset is immunological tolerance. More cytokines have been linked to ITP in recent years, according to research. The factors that can distinguish ITP from other types of thrombocytopenia and serve a specific role in the diagnosis of ITP. All immune-mediated thrombocytopenias, except for primary ITP, are classified as secondary ITP. 

Numerous diseases can cause secondary ITP, including autoimmune conditions such as systemic lupus erythematosus (SLE). SLE is a complex autoimmune illness that is frequently accompanied by hematological abnormalities [56], such as thrombocytopenia, which has been estimated to affect 7–30% of SLE patients [57,58]. It might be challenging to identify the kind of platelet reduction present in individuals with SLE in the initial stages when there are only thrombocytopenia symptoms [59]. Thrombocytopenia in SLE has a varied and complicated aetiology. However, it is generally acknowledged that the pathogenesis is aided by enhanced platelet clearance caused by platelet-specific autoantibodies, which is a mechanism similar to ITP [60].

There are 11 members of the interleukin (IL)-1 cytokine family of protein molecules, including IL-1 (IL-1F1), IL-1 (IL-1F2), IL-1 receptor antagonist (IL-1Ra, IL-1F3), IL-18 (IL-1F4), IL-36Ra (IL-1F5), IL-36 (IL-1F6), IL-37 (IL-1F7), and IL-36 (IL-1F8). The pathophysiology of SLE and ITP may be influenced by aberrant alterations in IL-18 and IL-18-binding protein (IL-18BP), according to several studies [61,62,63,64]. Furthermore, current research shows that IL-1 may contribute to the production of T-helper 17 (Th17) cells, which have been found to be more prevalent in individuals with SLE and ITP. This suggests that IL-1 may possibly have a role in inflammatory pathologies and autoimmune disorders [65,66].

In a study, IL-1 cytokines were measured in newly diagnosed ITP patients, SLE patients with thrombocytopenia (SLE-TP), SLE patients without thrombocytopenia (SLE-NTP), and healthy controls using a multiplex cytokine assay and RT-PCR [67]. In contrast to SLE-TP patients, SLE-NTP patients, and healthy controls, ITP patients had significantly lower serum levels of IL-1, IL-18, and IL-36. There was a favourable link between the platelet count and IL-37 level in ITP patients, despite the fact that there was no discernible difference in the serum level of IL-37 between ITP and SLE-TP patients. These findings suggested that blood levels of IL-1, IL-18, IL-36, IL-36 and IL-37 could serve as ITP biomarkers. As a result, blood levels of IL-1, IL-18, IL-36, and IL-36 could be used as biomarkers to distinguish SLE-TP patients from ITP patients [67,68].

These results deserve some comments. ITP and SLE-TP are both caused by antibodies that attack platelets; however, it is unclear what makes them different. Differences between ITP and SLETP patients’ peripheral blood absolute lymphocyte counts and neutrophil counts in this study point to distinct cellular immunity. IL-1, IL-18, IL-36, IL-36, and IL-33 are implicated in the aetiology of ITP but not SLE. The expression of IL-1 mRNA between ITP patients and SLE-TP patients did not differ significantly, which is an interesting finding. ITP and SLE-TP patients’ changes in IL-1 cytokine expression at the mRNA level did not match those seen at the protein level. Thus, it might be possible to infer from these findings that microRNAs and other post-transcriptional regulatory mechanisms may contribute to the pathophysiology of ITP and SLE-TP patients. 

Furthermore, in ITP patients, several other cytokines were shown to be changed. Interleukin (IL)-2 and interferon (IFN)-gamma levels were found to be higher in the serum, whereas IL-4 levels were noticeably lower. Thrombopoietin (TPO) levels have also been found to be normal, while increased levels of IL-11 have been noted [69]. These findings show that the Th1 type of T helper cytokine response is linked to ITP, but the Th2 type is downregulated. Megakaryocytes are initially found in bone marrow aspirates at normal quantities, which explains why TPO production is unaltered. The increased production of platelets per megakaryocyte may be a reflection of the rise in IL-11. Therefore, the cytokine profile and lymphocyte populations appear to be characteristic in the various forms of thrombocytopenia and could constitute a valid support for the differential diagnosis of the various pathologies.

### 2.6. Noncoding DNA and ITP Diagnosis

Several data revealed that more than 90% of the human genome could not be translated into proteins. Noncoding RNAs (ncRNAs), including long noncoding (lnc) RNAs and microRNA (miRNAs), are crucial in the development of human disorders [70]. MiRNAs are a subclass of ncRNAs that target the 3-UTR of mRNAs to control gene expression and protein translation [71,72]. Previous research revealed that miRNAs were dysregulated and connected to the control of ITP. For instance, miRNA-99a expression was augmented in CD4+ cells [73], while miRNA-182-5p and miRNA183-5p expression was augmented in ITP. Furthermore, in ITP, TGFB1 and IL18 were downregulated and inhibited by miRNA130A [74]. MiRNA409-3p was similarly noted to be decreased in ITP samples at the same time [75]. Additionally, lncRNAs were linked to autoimmune disorders and their symptoms. Wang et al. discovered that the expression of the lncRNA TMEVPG1 was lower in ITP subjects with respect to samples from healthy control subjects [76]. In a different study, 1177 and 632 lncRNAs were shown to be significantly up- or down-regulated in ITP patients, as compared to normal samples [77].

In an experiment that examined many open-access datasets, including GSE43177 and GSE43178, it was discovered that ITP patients had 468 upregulated mRNAs, 272 downregulated mRNAs, 134 upregulated lncRNAs, 23 downregulated lncRNAs, 29 upregulated miRNAs, and 39 downregulated miRNAs. After that, authors created networks in ITP for the coexpression of lncRNA, miRNA-mRNA, and protein-protein interactions. A bioinformatics investigation revealed that these genes controlled several biological functions in ITP, including translation, cell-cell adhesion, ubiquitin-mediated proteasome degradation, and mRNA nonsense-mediated decay.

As a result, patients with ITP appear to have a particular profile of miRNAs, which may be helpful for a more accurate diagnosis. However, it is worth noting that most of the results reported have not been reproduced, which may be due to diverse blood sources, or different population sizes, as well as the use of different RNA isolation and identification procedures. Thus, standardized detection schemes are needed in the next years, comprising more sensitive miRNA detection techniques and quantitative analysis models, while a consensus on the clinical significance of a few targets is necessary for their use in clinical practice [78].

### 2.7. Gut Microbiota and ITP

Numerous autoimmune illnesses present an altered gut microbiota, which was even recognized as one of their aetiologies. The human gut is home to more than one thousand different types of bacteria, which are crucial to both health and sickness [79,80,81,82,83]. The gut is where over 60% of human immunity is controlled. Mice raised in a germ-free environment have a weak immune system, and their immune cells significantly diminish. Recent research has revealed a connection between changes in the gut microbiota’s composition and functionality and illness symptoms, severity, and treatment response [84]. The therapeutic benefit of probiotic supplementation or fecal microbiota transplantation in individuals with autoimmune disorders is significant [85]. Additionally, several extraintestinal autoimmune diseases and immunological disorders, such as rheumatoid arthritis, type 1 diabetes, multiple sclerosis, and systemic lupus erythematosus, have been linked to gut microbiota [86]. 

The gut microbiome may also influence ITP. In a study, the metabolite profiles and gut microbial community were examined using feces from adult primary ITP patients who were untreated and healthy controls (HCs) [87]. According to the findings, ITP patients have lower levels of *Bacteroides* and higher levels of the fecal bacteria *Blautia*, *Streptococcus*, and *Lactobacillus*. Notably, fecal metabolites such as glycerophospholipids and fatty acids are enriched and intensely correlate with discrepant gut microbiota. 

*Weissella* and *Streptococcus anginosus*, Cer (t18:0/16:0), Cer (d18:1/17:0), and 13-hydroxyoctadecanoic acid mixtures may also be effective diagnostic indicators for ITP [87]. In conclusion, compared to HCs, ITP patients experience dysbiosis of the gut microbiota and metabolome. Several gut chemicals and bacteria changed by ITP can serve as ITP diagnostic biomarkers.

### 2.8. Complement Activation Biomarkers

An essential mechanism of platelet destruction is complement activation caused by anti-GPIIb/IIIa [88,89,90,91,92,93,94,95]. The complement system, a mostly blood-born protein cascade, has its evolutionary roots in homeostasis and innate immune protection [96]. ITP has been linked to shady levels of platelet-associated complement [97,98,99,100]. However, there have only been a few reports of studies on the regulation and specificity of complement activation [101].

In ITP patients with anti-GPIIb/IIIa antibodies, complement activation and improved complement activation capacity (CAC) were found both in vivo and in vitro studies. Patients in this group demonstrated decreased plasma levels of 2-GPI, which was negatively linked with C5b-9 deposition. Approximate physiological quantities of 2-GPI suppressed C5b-9 production in a dose-dependent manner [102] (Table 1). Inhibition of C3a production by β2-GPI and the presence of β2-GPI/C3 complexes in plasma suggested a control on the level of the C3 convertase. Additionally, c-Jun N-terminal kinase (JNK) phosphorylation levels were downregulated by 2-GPI, as was the cleavage of the BH3 interacting domain death agonist (Bid), which led to platelet lysis. These data suggest a unique relationship between decreased plasma 2-GPI levels and increased complement activation, suggesting that 2-GPI may be useful as a diagnostic biomarker.

Finally, anti-complement 1q antibody (anti-C1q), complement factor H (CFH), complement fragments Bb (CFBb), stromal-derived factor-1 (SDF1, also known as CXCL12), and IL21 plasma levels were examined by Sahip et al. to see if there was any correlation between them and the clinical characteristics of ITP. Patients with ITP had lower levels of CFH and CFBb and greater levels of anti-C1q compared to controls. The alterations in and CFH levels following treatment support the idea that the complement system plays a role in the pathogenesis of ITP [103].

**Table 1:** Possible biomarkers in ITP diagnosis.

Diagnostic Value	Biomarker	Significance	Refs.
	GPVI, GPIb/IX, and GPIIb/IIIa autoantibodies	Expression of autoimmune disease	[9,10,11,12]
	Reduction in the expression of FC gamma receptors (FCGR) IIb in macrophages	Correlation with H. Pylori infection	[13]
	Immature platelet fraction (IPF)	Ability to distinguish between thrombocytopenia due to consumptive processes platelet hypoproduction	[23,28,29,30,31,32,33,34,35,36,37]
	Expression of TRAIL in megakaryocytes	Megakaryocyte maturation index	[38]
	CXCR4	Maturation index	[42]
	CXCL13	Effect on immune response	[46,47]
	BAFF	Effect on the development of autoreactive B cells	[55]
	IL-1, IL-18, IL-36, IL-36, and IL-33	Ability to distinguish between primary and secondary thrombocytopenia	[67]
	IL-2, IL-11, IFN	Markers of Th1 type of T helper cytokine response	[69]
	miRNA-99, miRNA-182-5p, miRNA183-5p, miRNA130A, miRNA409-3p expression	Epigenetic control of cell-cell adhesion, ubiquitin-mediated proteasome degradation, and mRNA nonsense-mediated decay	[73,74,75]
	lncRNA TMEVPG1	Epigenetic control	[76,77,78,79]
	Gut microbiome	Effects on immune response	[87]
	Complement	Complement activation caused by anti-GPIIb/IIIa	[101,102]

### 2.9. Transcriptome Analysis

Finally, research that uses genetic analysis to find novel diagnostic indicators in ITP patients is extremely encouraging. The greatest number of platelet transcriptome samples were collected in a recent study. Using RNA sequencing (RNA-seq) transcriptomes, a thorough process of feature selection, feature engineering, and stacking classification was conducted to find the ITP biomarkers [104]. The final ITP detection model was trained using the 40 discovered biomarkers, and its overall accuracy was 0.974. The biomarkers revealed that a number of transcribed elements, such as protein-coding genes, long intergenic non-coding RNA genes, and pseudogenes with apparent transcriptions, may be linked to the start of ITP. The provided ITP detection model can also be used to diagnose ITP.

Numerous biomarkers displayed expression patterns that were highly tissue-specific; for example, the genes DNAH7 and AANAT were only strongly expressed in the testis, whereas the gene KLHDC8A was only highly expressed in the ovary. DNAH10OS, NORAD, MT-ATP8, HNRNPUL2, MT-RNR2, and MT-CO2 were among the genes with high expression in various brain regions, although the majority of the 40 biomarkers had relatively low expression in the total blood. It is critical to look into the molecular processes of ITP employing platelet cells since the data revealed that the abnormal expressions of these tissue-specific expressed genes may have contributed to ITP’s development and progression when combined with their ITP-specific expression patterns.

## 3. Prognostic Biomarkers: Refractory ITP

Even after receiving many lines of single-agent medications, some ITP patients continue to not react to conventional treatments, despite therapeutical advancements. Refractory ITP is linked to a severe decline in quality of life and extremely challenging therapeutic care. To make matters even more difficult, clinicians’ experience is crucial to properly treating refractory ITP because the diagnosis is still based on exclusion [105].

About 10% of ITP patients become resistive to treatment within a year, according to Psaila et al. [7]. In these situations, the lack of a clinical response calls into serious doubt the diagnosis of ITP [106] and should prompt a thorough clinical and laboratory work-up [107] to rule out other underlying illnesses, particularly myelodysplastic syndromes, drug-induced thrombocytopenia, inherited thrombocytopenia, and bone marrow failure syndromes. Additionally, type IIB von Willebrand disease and pseudothrombocytopenia should be ruled out.

Refractory ITP has been defined in several ways over the years. Refractory ITP used to typically be predicated on the absence of response or relapse following splenectomy. More specifically, failure to reach a platelet count of 30,000/L and a doubling of baseline platelet counts were used by Rodeghiero et al. to determine response [108]. The 2010 ASH guidelines [109] affirmed and supported this description of refractory ITP. However, splenectomy is not an option for a sizeable percentage of ITP patients, especially those who are elderly or have other serious comorbidities. Additionally, people may be reluctant to have a splenectomy and decline the treatment. Additionally, its pediatric indication is poor [110]. Cuker et al. expanded the definition of refractory ITP to include patients who need treatment but are unable or unwilling to have a splenectomy [111], while a complete lack of response to one or more single-agent treatments, such as rituximab and TPO-RA, was the definition of refractory ITP in 2020 [20]. Shortly after, Miltiadous et al. defined “refractory” patients as those whose platelet counts do not respond to more than two treatments, and whose platelet counts are extremely low and are accompanied by haemorrhage [112].

Therefore, it is clear how important it is to have accurate indicators that can forecast how the disease will progress. Insidious onset, a higher platelet count at presentation, female gender, older age at presentation, a lack of prior infection or vaccination, positivity for antinuclear antibodies (ANA), and an inability to respond to a single dose of intravenous human immunoglobulins are all thought to be predictors of the chronic course of the disease.

### 3.1. Genetic Characteristics in Refractory ITP Patients

According to some results, children with chronic ITP have a strong family history of the condition [113]. Additionally, there is proof that ITP is inherited, with some immune-related genes perhaps playing a role [114]. The clinical characteristics and genetics of chronic refractory immune thrombocytopenia (C/RITP) in infants, as well as their significance in treatment refractoriness, have, however, received very little research attention.

In a study, children with C/RITP who had immune-related gene mutations were examined for their clinical symptoms and genetic traits [115]. Children in the mutant group had more severe hemorrhages, more aberrant immunological indices, and greater levels of SLE biomarker expression. The mutant group’s peripheral T and B lymphocyte counts dramatically increased. *TNFRSF13B*, *CARD11*, *CBL*, and *RAG2* are four genes linked to primary immunodeficiencies which are mutated in 17.6% of patients, while 23 other genes had variants in 82.4% of patients that were of unknown importance [115].

The mutant group’s elevated risk of several aberrant immunological phenotypes could be a sign of a hereditary propensity for immunodeficiencies. Immune problems manifested early in the mutation group of patients. Theoretically, this implies that they require more frequent immunosuppressive therapy and the utilization of second-line therapies, and that the prognosis for these kids is worse. 

The inflammasome complex was subjected to a separate genomic investigation. The well-studied inflammasome NLRP3 (NOD-like receptor pyrin domain-containing protein 3) is a component of the innate immune system that reacts to cellular stress by releasing the proinflammatory cytokines IL-1 and IL-18. Numerous inflammatory and autoimmune illnesses, including diabetes, obesity, and atherosclerosis, are triggered by the NLRP3 inflammasome [116,117].

ITP patients’ gene expression and polymorphisms for the NLRP3 inflammasome were examined using RT-PCR [118]. By using flow cytometry, T helper cells and apoptosis of peripheral blood mononuclear cell (PBMC) from ITP patients were examined. The NF-B-94ins/del ATTG genotype was found to contribute to ITP susceptibility, according to the results. Additionally, ITP patients with the WW genotype or WD genotype had lower platelet counts than ITP patients with the DD genotype of NF-B-94ins/del ATTG. ITP patients with the WW or WD genotype showed higher mRNA expression than those with the DD genotype when compared to controls for NF-B gene expression. Similar to this, the WW genotype also showed enhanced NLRP3 mRNA expression. In the group that was not stimulated, there was no discernible change in the percentage of Th17 cells for the genotypes WW, WD, and DD (WW WD DD), although there was a substantial gene dosage effect. In ITP patients, activation of the NLRP3 inflammasome may upregulate Th17 [118]. In summary, the NF-B-94ins/del ATTG genotype may be a new biomarker and possible target for ITP.

### 3.2. Circulating Microparticles as Prognostic Markers

Circulating microparticles (MPs), which are extracellular vesicles (EVs) that cells release in response to activation or stress, are an alternative, potential biological marker. MPs carry particular sets of proteins, lipids, and RNAs that may serve as a communication medium between cells, depending on their biological origins [119,120]. The glycoproteins CD41 and CD42b as well as phosphatidylserine are some of the surface indicators that platelet-derived microparticles (PMPs) and megakaryocyte-derived microparticles (MKMPs) have in common. However, PMPs can be separated from MKMPs by the expression of CD62P [121].

In the meantime, higher PMP levels have been linked to ITP and have been reported to give some ITP patients procoagulant characteristics [122,123]. It has been discovered that MKMPs, which are generated during megakaryocyte maturation [124], can stimulate platelet formation without the need for additional TPO [125,126]. According to these results, MKMPs could be used as biomarkers to monitor megakaryocyte dynamics and as a potential ITP treatment. Megakaryocyte maturation in ITP patients has also been found to be hindered in previous bone marrow smear and ultrastructural examinations [127].

In numerous autoimmune disorders, altered cell-derived microparticles (MPs) have been seen. However, little research has been performed on the functions of MKMPs and PMPs produced from megakaryocytes and platelets in ITP. Researchers analysed plasma MKMP and PMP levels in ITP patients and assessed the studies’ potential diagnostic utility [128]. In a discovery set of ITP patients, non-immune thrombocytopenia (TP) patients, and age- and gender-matched healthy controls, plasma MKMP and PMP levels were examined by flow cytometry. The effectiveness of the thrombopoietin receptor agonist (TPO-RA) therapy was evaluated using samples from a therapy group of ITP patients. The findings showed that plasma MKMP and PMP levels were greater in TP patients but significantly lower in ITP patients compared to healthy controls. The PMP/platelet ratios in ITP patients were higher than those in TP patients and healthy controls after normalization to platelet counts. PMP/platelet ratios had a 73.1% sensitivity and 77.3% specificity for diagnosing ITP. With a sensitivity of 74.4% and a cut-off value of 112.5 MPs/L, MKMP levels can be utilized to distinguish between ITP and TP. In addition, ITP patients who responded to TPO-RA treatment had higher MKMP and PMP levels [128]. According to these findings, plasma MKMP and PMP levels are lower in ITP patients, and they are also new prognostic biomarkers for ITP.

### 3.3. Telomere Length and Platelet Replication Ability

Finally, prognostic markers that can reveal a cell’s capacity for reproduction may be valuable. The repeating TTAGGG repeats and accompanying proteins, collectively known as the shelterin complex, make up the specialized DNA-protein structures known as telomeres, which are found at the ends of linear chromosomes in eukaryotic cells. The cellular DNA-repair machinery uses the shelterin complex as a signal to discriminate between telomeres and DNA double-strand breaks [129,130]. Thus, the telomeres play a role in replication as well as the preservation of genomic and cellular stability [131]. In fact, there is an “end-replication problem” because ordinary DNA polymerases are unable to properly copy the ends of a linear DNA molecule, causing telomere shortening with each cell division. Telomere length can therefore be seen as a measure of cell proliferation history and lingering potential for replication [132,133,134]. Additionally, lymphocyte telomere length can affect immunological response [135]. Telomere shortening in lymphocytes is thought to signify immune system aging and may be a risk factor for autoimmune reactions [136].

Comparing CD4+, CD8+, and CD19+ lymphocytes from ITP patients to those from healthy controls, telomerase activity was shown to be higher [137]. In ITP patients, there was a slight negative connection between platelet count and telomerase activity of CD19+ cells. In comparison to the healthy controls, the relative telomere length of PBMC in ITP patients was considerably shorter.

Telomere length of PBMC was shown to be considerably shorter in active ITP patients compared to controls, and it also tended to be shorter in inactive ITP patients [137]. In addition to the initial platelet count and the severity of the bleeding, telomere length is an independent predictor for the prognosis of ITP in this study. Patients with lower telomere lengths are more likely to get chronic ITP. On the other side, people with ITP who have telomeres that are longer typically have better prognoses.

## 4. Response to Specific Treatment

High-dose glucocorticoids are advised as the first-line treatment for adult ITP patients, according to consensus clinical recommendations [107]. Clinical problems, however, include frequent side effects and varied responses, with 20–30% of patients failing to react at safe levels [138]. Mycophenolate or rituximab may be added to glucocorticoids to boost response rates, although these drugs have lower a quality of life and more side events [139,140]. Predicting which patients are likely to fail glucocorticoid monotherapy and who would benefit from early supplemental treatment would therefore be clinically useful.

When taking the immunological aetiology of the pathology into account, it is clear that a particular cytokine composition and a certain profile of the cell populations may be suggestive of a different response to immunosuppressive therapy. For instance, in the absence of the anti-inflammatory cytokine IL-10, cells that express the pro-inflammatory cytokines IL-17 and IFN-c are resistant to inhibition [141,142]. Additionally, activated CD4+ T cells from glucocorticoid-refractory ITP patients demonstrated a relative abrogation of IL-10 with persisting IL-17 in response to in vitro glucocorticoids compared to responsive individuals [143].

Furthermore, numerous cytokine gene polymorphisms, such as certain IL-10 and IFN- genotypes, have been linked to the efficiency of corticosteroid therapy in ITP [144,145].

CD4+ T cells from ITP patients were cultured in the presence or absence of dexamethasone (Dex) in a prospective cohort study [146]. The clinical response of the patients to corticosteroid therapy was then compared with intracellular cytokine expression. To determine whether findings were specific to ITP or if they might be applicable to other autoimmune disorders, a control cohort of patients with autoimmune uveitis was also investigated. Following CD4+ T cell culture with Dex, the ratio of IL-10 to IL-17 expression was able to distinguish between ITP patients with a clinically defined full, partial, or nonresponse to corticosteroid treatment. Patients with autoimmune uveitis confirmed these findings [146]. As a result, IL-10 expression has decreased somewhat, but IL-17 expression has persisted in the CD4+ T cells of subjects who clinically fail steroid treatment. This finding may help us better understand how corticosteroids work to treat ITP and serve as a biomarker for steroid-resistant disease, with potential applications to a variety of hematological and nonhematological disorders.

Another study found that non responder (NR) patients’ ex vivo CD4+ T cells had a decreased IL-10:IL-17 ratio. Results from samples that were followed up after two months support this conclusion [147]. This implies that CD4+ T cells from NR patients tend to produce higher levels of IL-17 and lower levels of IL-10 over time. The stimulation of IL-10 in a variety of immune cell types, such as CD4+ and CD8+ T cells, as well as B cells, is most likely a crucial factor in the effectiveness of glucocorticoids [148,149,150]. The discovered ex vivo T cell profile might be transformed into a clinically useful biomarker to help identify NR patients, which could then guide the clinical choice to start alternative therapy early.

### 4.1. Desialylation and Glycosylation as Indicators of Response

Antibody specificity (i.e., anti-GPIIbIIIa versus anti-GPIb-IX) may play a significant role in dictating the response to therapy in ITP, with the presence of anti-GPIb-IX antibodies resulting in a decreased response to corticosteroids and IVIG, according to murine models and large cohort human studies published in recent years [151,152,153]. Most recently, a study found that anti-GPIb and some anti-GPIIbIIIa antibodies in humans caused platelet desialylation, which then led to Fc-independent platelet clearance in the liver through hepatic asialoglycoprotein Ashwell-Morell receptors [154]. This finding raises the possibility that antibody-mediated desialylation may be one of the underlying mechanisms behind resistance to steroid and intravenous immunoglobulin G treatment [155].

The removal of the terminal sialic acid residues from glycans, which starts the catabolism of glycans, modifies the structure and functions of glycans, glycoproteins, or glycolipids. Desialylation is a crucial component of sialic acid metabolism. The roles of sialic acids are well understood, while those of the desialylation process are either poorly understood or completely neglected. Nevertheless, mounting proof shows that desialylation is crucial for several physiological and pathological activities.

Recent research has revealed a unique Fc-independent platelet clearance pathway, in which antibody-mediated desialylated platelets can be removed in the liver via asialoglycoprotein receptors, decreasing the response to first-line treatments that target Fc-dependent platelet clearance [156]. The study compared the levels of platelet desialylation with the effectiveness of first-line therapy to assess the importance of this result in ITP patients. They discovered that there was a statistically significant difference in desialylation levels between various treatment response groups. Importantly, correlation analysis showed a relationship between treatment response and platelet desialylation, with non-responders having considerably greater platelet desialylation levels. Interestingly, they discovered substantial platelet desialylation in secondary ITP and some non-ITP thrombocytopenias, as compared to healthy controls [156]. According to these findings, platelet desialylation is a crucial biomarker for assessing how ITP responds to conventional therapy. 

As for the mechanism, at asparagine 297, the Fc component of IgG has a distinctively conserved glycosylation site (Asn297). Through the alteration of the IgG Fc binding affinity for Fc receptors (FcRs) and the complement protein C1q complex, the precise composition of the attached N-glycan influences IgG-mediated effector functions such as antibody-dependent cell-mediated cytotoxicity (ADCC), complement-dependent cytotoxicity (CDC), and antibody-dependent cellular phagocytosis (ADCP) [157]. Inflammatory and autoimmune illnesses may be exacerbated by the glycosylation of IgG Fc, which is essential for controlling IgG’s pro- and anti-inflammatory actions. Numerous autoimmune and inflammatory conditions, including rheumatoid arthritis, systemic lupus erythematosus, inflammatory bowel disease, and autoimmune hemolytic anemia, have been shown to have skewed IgG glycosylation patterns [158,159,160,161]. In some cases, the skewed IgG glycosylation is linked to the severity of the condition and how well it responds to treatment [162,163].

In a study, the N-glycan profiles of serum proteins and the purified IgG fraction were compared between ITP patients and healthy controls. The study also looked into the relationships between N-glycans and platelet counts, and found that ITP patients had unique N-glycan patterns in both their serum and IgG [164]. Serum fucosylation, IgG galactosylation, six of twelve of serum N-glycan peaks, and six of seven IgG N-glycan peaks were all substantially different between ITP patients and healthy controls. IgG peak seven demonstrated good diagnostic performance in separating ITP patients from healthy people. Serum fucosylation was considerably reduced in ITP patients with severe thrombocytopenia compared to ITP patients with mild and moderate thrombocytopenia. In ITP patients with severe thrombocytopenia, serum fucosylation and serum peak five were linked with platelet counts, and the absolute values of the correlation coefficients were both over 0.5 [164]. Patients with ITP showed distinct N-glycan patterns in their serum and IgG. A potential biomarker for supplementary ITP diagnosis was IgG peak seven.

A different study investigated the role of Asn279-linked N-glycan of auto antibodies in vitro and in vivo. AAb-induced platelet phagocytosis was inhibited by N-glycan cleavage. Injection of AAbs resulted in the rapid clearance of human platelets compared to control. Auto antibodies that were able to activate complement induced more pronounced platelet clearance in the presence of complement compared to the clearance in the absence of complement. Auto antibodies lost their ability to destroy platelets in vivo after deglycosylation. N-glycosylation of human ITP auto antibodies appears to be required for platelet phagocytosis and complement activation, reducing platelet survival in vivo. Posttranslational modification of auto antibodies may constitute an important determinant for the clinical manifestation of ITP [165].

Moreover, the relationships between n-glycan and other molecules, such as haptoglobin, appear to be particularly interesting. In fact, altered glycosylation patterns of plasma proteins are associated with autoimmune disorders and the pathogenesis of various diseases, and n-glycan can fine-tune Hp interactions [166].

### 4.2. TNF Receptor-Associated Factors

A study sought to assess lymphocyte attraction as a potential additional factor in the aetiology of ITP. The TNF superfamily and the Toll-like receptor (TLR) family are two families of adaptor proteins that have biological activities that include cellular death, survival, and immunological responses [167]. Through pathways such as TRAF6, signals from receptors including CD40, RANKL, and IL-1 have been conveyed. TRAF6 has been associated with several diseases, including myeloma and myelodysplastic syndrome, due to its role in attracting lymphocytes and transmitting inflammatory signals [168,169]. With the potential for utility in the therapeutic setting as a biomarker of corticosteroid or IVIG response, a study was conducted to assess the importance of TRAF6 as an immune-signaling component in the aetiology of ITP [170]. TRAF6 levels were different in the patient group and in the control group (2348 pg/mL vs. 25.57 pg/mL) and levels were lower in corticosteroid-responding patients than in nonresponding patients. This finding points to a direct connection between TRAF6 and the pathophysiology of ITP [170]. The understanding of TRAF6 in the antibody-mediated immune system did correlate with these findings. Corticosteroid responses were weak among patients with the highest TRAF6 levels, according to the investigators’ observations. Patients with the highest TRAF6 levels, on the other hand, reacted to IVIG more quickly. These connections suggested that TRAF6 and the degree of immunological reactivity in the context of the antibody-mediated system may be connected. As a result, it is possible to evaluate TRAF6 patients at the time of the initial presentation and forecast a response to corticosteroids as well as to other treatment modalities such as rituximab, IVIG, and splenectomy.

A different therapeutic option for ITP patients who do not react to glucocorticoid therapy or who need ongoing high-dose glucocorticoids to maintain a safe platelet count is splenectomy. According to the findings of various research, splenectomy is effective in about two thirds of adult patients and 70 to 80 percent of youngsters [108]. Splenectomy for ITP, however, is frequently linked to a high risk of serious morbidity and mortality, and the long-term haematological results of the treatment cannot be anticipated using commonly used indicators [171]. Younger age [172,173,174], prior response to steroids, and postoperative peak platelet count [175,176] have all been reported in several studies as potential predictors of a sustained response to splenectomy. However, the findings of other investigations were contradictory [177,178,179]. 

The location of autologous 111In-labeled platelet sequestration was discovered to be a reliable prognostic factor by Najean et al. [180]. However, isotope assessment methods are frequently qualitative rather than quantitative, and splenectomy is effective for many individuals with non-splenic sequestration.

Hepatocytes in the liver produce most of the acute phase protein known as haptoglobin (HP). IL-6, which is created by the key cytokines TNF- and IL-1, is the main factor that stimulates the expression of Hp [181]. Serum levels of Hp are usually quite stable; hence, finding a noticeable variation in serum Hp expression has clinical importance [182]. Hp is made up of two polypeptide chains. Only one kind of chain exists, and it has a MW of roughly 40 kDa. Two isoforms of the chain, however—designated as -1 and -2—represent the chain; the MW values for -1 and -2 are, respectively, 16 kDa and 9 kDa [183]. 

Recent proteomic research demonstrated the significance of Hp as a biomarker in a few autoimmune disorders. The cerebral fluid of patients with Guillain-Barré syndrome, an acute inflammatory autoimmune illness of the peripheral nerve system, has elevated amounts of the protein Hp. According to other investigations, systemic lupus erythematosus and active Behcet’s illness both have increased disease activity when there are raised serum levels of Hp [184,185].

In one investigation, serum samples from ITP patients were taken both before and seven days after the splenectomy [186]. Pooled preoperative serum samples from patients who responded to splenectomy, patients who did not respond, and healthy controls were subjected to two-dimensional gel electrophoresis following the depletion of the abundant serum proteins. By using matrix-assisted laser desorption/ionization time-of-flight mass spectrometer analysis, nine protein spots with at least a five-fold difference in expression between responders and non-responders were all identified as Hp. Thirty-seven responders, thirteen non-responders, and twenty-one healthy controls underwent enzyme-linked immunosorbent tests to validate the expression of serum Hp. The non-responders had considerably lower preoperative serum levels of Hp than the responders. The preoperative platelet counts and preoperative serum levels of Hp did not significantly correlate; however, they did positively correlate with postoperative peak platelet counts. The receiver operating characteristic curve’s best cut-off value for preoperative serum Hp levels (1173.80 g/mL) resulted in 78.4% sensitivity and 84.6% specificity [186] (Table 2).

Hp has been proven to modulate both innate and adaptive immune responses in several aspects. Th1 and Th2 play a crucial role in the pathogenesis of autoimmune diseases. Studies performed both in vitro and in vivo indicate that Hp exhibits a significant modulating impact on the Th1/Th2 balance via an inhibitory effect on Th2 cytokine release and therefore promotes a dominant Th1 cellular response. In a study, HP was identified as a potential serum biomarker, which may serve as a major predictor of the long-term response to splenectomy in ITP patients [186]. These findings imply that serum Hp levels could be a useful predictor of the long-term success of splenectomy in ITP and could shed light on the pathophysiological distinctions between responders and non-responders.

**Table 2:** Prognostic and treatment response biomarkers.

Prognostic Value	Biomarker	Significance	Refs.
	Gene mutations (*TNFRSF13B, CARD11, CBL,* and *RAG2)*	More severe haemorrhages, more aberrant immunological indices, and greater levels of SLE biomarker expression	[115]
	Polymorphism for the NLRP3 inflammasome	Lower platelet counts	[118]
	Platelet-derived microparticles and megakaryocyte-derived microparticles	Procoagulant characteristics	[122,123,128]
	Telomere length and telomerase activity	Autoimmune reactions, correlation with platelet counts and severity of the bleeding, correlation with outcome.	[136,137]
Response to treatment	Cytokine composition and cell population profile	Response to glucocorticoids	[141,142,143,147]
	Cytokine gene polymorphisms	Response to glucocorticoids	[144,145]
	Platelet desialylation	Response to glucocorticoids	[155,156]
	TRAF6 levels	Response to glucocorticoids and immunoglobulin treatment	[170]
	Age, response to steroids, and postoperative peak platelet count	Response to splenectomy	[172,173,174,175,176]
	Haptoglobin	Response to splenectomy	[186]

## 5. ITP and Oxidative Stress

Finally, given the wide range of consequences that this topic entails, a distinct section should be set aside to discuss the connection between ITP and oxidative stress.

Free radicals are molecules or molecular fragments that have one or more unpaired electrons in atomic or molecular orbitals and are highly reactive [187]. Reactive oxygen species (ROS) are by-products of normal cellular oxygen metabolism by enzymatic reactions, such as the mitochondrial respiratory chain, nitric oxide synthases, xanthine oxidase, or NADPH oxidases [188]. ROS can also result from exposure to extracellular stressors such as radiation and inflammatory cytokines. The majority of a cell’s mitochondria produce the superoxide anion radical, which can then combine with other molecules to produce secondary ROS such as hydrogen peroxide and hydroxyl radicals [189]. Through the generation of peroxides and free radicals that harm all cell components, disturbances in this normal redox state can have harmful effects [190]. Antioxidants and antioxidant enzymes including catalase (CAT), glutathione peroxidase (GPx), and superoxide dismutase (SOD) are in charge of controlling this redox state to prevent cellular damage [191].

A biological system’s inability to quickly detoxify the reactive intermediates or quickly repair the damage that results from ROS generation is what leads to oxidative stress [189]. 

Diet and regular lifestyle play a role in maintaining well-being and preventing diseases. In recent years, due to their potential antioxidant activity, the use of polyphenols has increased as a barrier to ROS [192]. This class of ROS-reactive molecules are organic compounds that are abundant in many vegetable species. In recent years, scientific studies on these bioactive molecules have intensified, demonstrating that the consumption of polyphenols can contribute to the control of ROS in the physiological functions of our body [193].

In recent years many food products enriched with antioxidant substances have been created to block free radicals. In fact, the European Food Safety Authority (EFSA) has stated that the consumption of 5 mg/kg/day of polyphenols could help in the prevention of such diseases connected to the ROS [194].

Numerous hematological diseases have been found to disrupt the oxidative balance [195,196,197,198,199]. In addition, oxidative stress and autoimmune illnesses have a long history of association. Aldehyde-modified proteins have a high immunogenicity. When animals are inoculated with oxidized low-density lipoprotein (LDL) particles, autoantibodies directed against the epitopes of LDLs modified by malondialdehyde (MDA) and 4-hydroxynonenal (HNE) are formed [200]. The oxidative alteration of protein antigens may modify the adaptive immune response. In numerous autoimmune illnesses, including scleroderma, Behcet’s disease, systemic lupus erythematosus, rheumatoid arthritis, and type 1 diabetes mellitus, the oxidative alteration of proteins has been demonstrated to generate pathogenic antibodies [201]. Additionally, protein changes brought on by free radicals are highly immunogenic and can cause an antibody response by activating the adaptive immune system [202]. Regulatory T cells (Tregs) play a crucial role in self-tolerance, and a number of studies [203,204,205,206] have shown that abnormalities in Tregs lead to increased T-cell and B-cell autoreactivity in individuals with ITP. It is interesting to note that in autoimmune illnesses, NO was demonstrated to diminish Foxp3 expression and, consequently, Tregs [207]. As a result, ROS may be a factor in the Treg shortage in ITP, and redox modulation may also be involved in immune regulation.

All these findings suggested that increased oxidative stress may be a significant factor in the pathogenesis of ITP and in the destruction of the platelet membrane, which results in the loss of cell membrane elasticity, an increase in cell fragility, and a shortening of cellular life. 

Several studies have discovered significant oxidative state changes in ITP patients. An analysis of the blood expression profiles of ITP patients and healthy individuals revealed that ITP patients had activated ROS-related molecular signaling pathways. 

Moreover, an unbalanced redox state results from excessive ROS production or insufficient antioxidant scavenging capacity in ITP patients. A smaller ratio denotes a higher level of oxidative stress, and the ratio of reduced to oxidized glutathione (GSH/GSSG) serves as an excellent indicator of the oxidative stress status. The value of this marker is even lower in patients with chronic ITP than in those with acute ITP, and it is significantly higher in healthy controls than in ITP patients [208,209]. 

As indicated by higher MDA levels in the plasma of ITP patients compared to controls, persistent oxidative stress results in lipid peroxidation and the formation of reactive aldehydes [210]. Protein carbonylation is a sort of post-translational alteration of proteins that results from either the direct oxidation of amino acid residues or the indirect oxidation caused by the addition of aldehydes (more common). In contrast to native human serum albumin (HAS), which only produced low-titre antibodies, HAS transformed in vitro by hydroxyl radical was found to be a strong antigenic stimulus, eliciting high-titre antibodies in rabbits [211]. The creation of neo-epitopes may be the cause of the ROS modified HSA’s much increased immunogenicity. Given that oxidatively changed proteins have been found to be highly immunogenic, it is conceivable to hypothesize that the development of autoantigen in ITP may have been triggered by a similar process, at least in part.

Additional research appears to support the significance of oxidative stress in the pathophysiology of ITP and the potential use of oxidative stress markers in the diagnosis, prognosis, or perhaps as an effective treatment target. For instance, significantly increased levels of MDA, NO, and oxidized glutathione were found in a cohort with chronic ITP in observational research. Additionally, it was discovered that these individuals with chronic ITP had considerably reduced levels of CAT, TAC, and superoxide dismutase [212]. 

The pathophysiology of ITP was therefore hypothesized to involve both increased oxidative stress brought on by inflammation and decreased antioxidant levels [213,214,215,216,217].

Moreover, a genetic biomarker, the overexpression of vanin-1 (VNN-1), an oxidative stress sensor, has been proposed as a predictor of chronic disease, and other markers have been found with prognostic value [218,219]. A copper and zinc deficiency results in oxidative stress because they are components of the antioxidant system. The response to first-line therapy for primary immune thrombocytopenia and the serum copper level were found to be significantly correlated, while in relapsed patients, the serum copper level was considerably lower [220].

High-mobility group box 1 (HMGB1), a distinct redox-sensitive protein, is thought to have a role in controlling stress reactions to oxidative damage and cell death. This nuclear non-histone protein regulates chromosomal structure and function by acting as a DNA chaperone. As a damage-associated molecular pattern protein, HMGB1 can also be released into the extracellular environment and operate during several types of cell death, such as apoptosis, necrosis, pyroptosis, necroptosis, alkaliptosis, ferroptosis, and cuproptosis. After being released, HMGB1 interacts with membrane receptors to influence metabolic and immunological responses. The redox status and protein posttranslational modifications of HMGB1, as well as its subcellular location, affect its function and activity [221].

Several studies showed that the etiology of autoimmune disorders and malignancies is influenced by abnormal HMGB1 [222,223]. Rheumatoid arthritis patients had greater amounts of HMGB1 in their synovial fluid, serum, and synovial tissue. HMGB1 may also play a role in the progression of primary Sjögren’s syndrome, systemic lupus erythematosus, and other autoimmune illnesses.

In an experiment, HMGB1 expression was shown to be considerably higher and Foxp3 expression to be lower in the spleen of patients with refractory ITP. Moreover, Foxp3 and HMGB1 were found to have a substantial negative connection, and HMGB1 overexpression was significantly linked with poor splenectomy efficacy. As for other immune parameters, IL-10 had a negative association with HMGB1, while HMGB1 expression increased and was positively linked with IL-17 in serum. The expression of HMGB1, RORt, and Foxp3 changed in PBMCs, with the alterations being more pronounced in the group with refractory chronic ITP. In a coculture system using PBMCs from untreated ITP patients, rHMGB1 elevated RORt expression and lowered Foxp3 expression, while an antiHMGB1 antibody partially reversed the aforementioned effects. These results imply that HMGB1 participates in the etiology of ITP and is linked to the imbalance of Treg/Th17 cells [224]. Additionally, this theory was supported by other investigations [225]. Evaluation of HMGB1 levels could be a useful means of monitoring the progress of the disease.

Finally, as was already indicated, modulating oxidative stress may be important for treating ITP. Patients with ITP who have measurable oxidative stress may benefit from adjuvant antioxidant therapy to increase platelet count. According to a study, antioxidant therapy reduced oxidative stress in the ND and chronic ITP groups, which may have contributed to improvements in the bleeding score and platelet count [217]. Probably because of its anti-inflammatory effects in ITP patients with severe clinical symptoms necessitating medication, high-dose methylprednisolone therapy lowers oxidative stress [226]. Similar to this, Brox et al. reported on the successful use of the well-known antioxidant ascorbic acid in the treatment of a small number of adult patients with refractory ITP [227]. However, several small-scale studies were conducted in the past to determine whether ascorbic acid was effective in treating chronic ITP, and the results were highly debatable [228,229,230,231,232].

Currently, six different trials are underway to evaluate the efficacy of antioxidant therapy in the treatment of ITP [233] (Table 3).

Finally, oxidative stress biomarkers could be useful for predicting therapeutic success. It was discovered that the average levels of native and total thiol in the patient group were substantially lower than those of the controls. However, IVIG therapy stopped these declines. Disulfide levels were marginally but not significantly lower in ITP patients; however, they increased after IVIG therapy [234].

In conclusion, at this time, it would be reasonable to conduct large-scale prospective clinical trials to determine whether anti-oxidants are useful in treating patients with ITP.

## 6. Conclusions

Platelet counts and the exclusion of morphological abnormalities in peripheral blood smears are the main diagnostic criteria for the diagnosis of TP, a common bleeding condition. Without a trustworthy biomarker, incorrect diagnoses are frequent and can result in excessive bleeding incidents, patient anguish, exposure to inappropriate drugs, and the need for invasive treatments such as splenectomy. However, over the past few years, a number of biomarkers have emerged that could be used to diagnose ITP, predict prognosis, and gauge therapy effectiveness.

A clinical prediction model (CPM) for the diagnosis of ITP was recently created to assist clinicians in examining patients who come with undifferentiated thrombocytopenia [235]. Based on information from patients with thrombocytopenia registered in the McMaster ITP registry, the authors created the Predict-ITP Tool, a CPM for ITP diagnosis at the time of the initial hematological consultation. A platelet count of fewer than 100 109/L and a platelet count response following high-dose corticosteroids or intravenous immune globulin were used to characterize ITP cases. A platelet count response was defined as the achievement of a platelet count of more than 50 109/L and at least a doubling of baseline. Bootstrap resampling was used for the internal validation. The c-statistic was used to evaluate model discrimination, while the calibration slope, calibration-in-the-large, and calibration plot were used to evaluate calibration. The final model had the following variables: severe bleeding history; lowest platelet count value; highest mean platelet volume; platelet count variability (based on three or more platelet count values); and lowest platelet count value (defined by the ITP bleeding scale). The optimism-corrected c-statistic was 0.83, the calibration slope was 0.88, and the calibration-in-the-large was 0.001 with a standard error of 0.001 for all performance metrics, showing excellent calibration and good discrimination. For a specific patient with thrombocytopenia at the time of the initial haematology consultation, the Predict-ITP Tool can predict the chance of ITP [235]. The instrument exhibited a high degree of prediction accuracy for ITP diagnosis. 

The use of various biomarkers to facilitate the diagnostic and prognostic evaluation of ITP patients was suggested by several experimental results, which is a promising advance that has undoubtedly raised interest in their application. However, it must be acknowledged that there are still some limitations since various analysers will need reference ranges to be established before application, and as technology develops and newer analysers with higher specificity and sensitivity become available, these ranges will undoubtedly need to be revised. Furthermore, it must be kept in mind that the different biomarkers do not have absolute significance in patients with ITP. It is highly likely that one candidate lower in early ITP pathogenesis may be elevated in chronic or later phases, or refractory ITP. For instance, in a study, plasma miRNAs were evaluated in patients with acute ITP (aITP) and chronic ITP (cITP). The detection of significant differences between plasma miRNA levels of aITP and cITP patients may provide useful information in the prediction of the course of disease, determination of disease etiopathogenesis, and the development of new therapeutic modalities [236]. Similarly, the frequencies of circulating B cells secreting platelet-specific antibody in acute ITP patients were notably increased compared to the chronic ITP patients [237], while both IL-2 and IFN-gamma were significantly increased in chronic ITP when compared to acute ITP and platelet associated IgM was detected more in acute than in chronic ITP [238].

Despite these apparent drawbacks, novel biomarkers may be useful for determining the cause of a thrombocytopenic presentation.

A greater understanding of the pathophysiology of ITP has made it possible to find new disease biomarkers that can aid in its detection. Some of the promising disease markers include the search for platelet autoantibodies, analysis of the transcriptome and complement activity, evaluation of oxidative stress markers, valuation of chemokines and non-coding genetic material, analysis of factors that can control the growth and activity of immunological effectors such as BAFF and cytokines, and assessment of the immature platelet fraction and megakaryocyte maturation index. How many of these might actually have therapeutic utility and how many of these have a positive cost-benefit profile need to be assessed in controlled research. In actuality, some of these tests seem pricey or technically challenging to carry out. It is possible that a score created by combining some of the investigated biomarkers could serve as an effective diagnostic tool. 

Finally, it is necessary that the assay techniques used for the evaluation of biomarkers improve in sensitivity and specificity. Most T cells in mammals, including humans, present the αβ T cell receptor and identify a specific peptide bound to a major histocompatibility complex molecule (pMHC) expressed on target cells [239]. The weak equilibrium dissociation constant between the TCR and monomeric pMHC causes a transient complex that obstructs easy identification [240]. Mallajosyula et al. engineered a biotinylation site on maxi-ferritin to generate a 24-subunit, self-assembling protein scaffold for the multivalent display of pMHC. This spheromer platform presents several advantages, including ease of production and defined site-specific conjugation of pMHC molecules that significantly decreases interbatch variation [241]. The introduction of this method could be useful to enhance the avidity of low-affinity interactions, and to boost detection of low-affinity autoantibody detection.

Last but not least, it is likely that in the future, research on additional blood markers of platelet activity [242] will yield novel indicators that will make it easier to diagnose the condition and accurately forecast how well it will respond to treatment.

## Figures and Tables

**Table 3:** Studies currently underway to evaluate the efficacy of antioxidant therapy in the treatment of ITP.

NCT Number	Title	Status	Condition	Interventions	Characteristics
NCT01763658	Oxidant Status and Effect of Antioxidant in Immune Thrombocytopenia	Unknown status	Immune Thrombocytopenia	Drug: Antox tablets (Mepaco) Other: drug therapy for ITP	Study Type: Interventional Phase: Phase 2 Phase 3
NCT03460808	The Combination of Atorvastatin, Acetylcysteine and Danazol as the Treatment of Steroid-resistant/Relapse Immune Thrombocytopenia	Unknown status	Immune Thrombocytopenia	Drug: Atorvastatin Drug: Acetylcysteine Drug: Danazol	Study Type: Interventional Phase: Phase 1 Phase 2
NCT04368598	The Combination of High-dose Dexamethasone and Acetylcysteine as the Treatment of Newly-diagnosed ITP	Unknown status	Immune Thrombocytopenia	Drug: Dexamethasone Drug: Acetylcysteine	Study Type: Interventional Phase: Phase 2
NCT05551624	Evaluation of the Effect in Platelet Count of Atorvastatin and N-acetyl Cysteine	Completed	Primary immune Thrombocytopenia	Drug: Atorvastatin 40 Mg Oral Tablet + N-acetylcysteine 400 mg Oral tablet	Study Type: Interventional Phase: Early Phase 1
NCT02351622	Caffeic Acid Tablets as a Second-line Therapy for ITP	Completed	Immune Thrombocytopenia	Drug: Caffeic acid Drug: Dexamethasone Drug: Placebo	Study Type: Interventional Phase: Phase 3
NCT02556814	Caffeic Acid Combining High-dose Dexamethasone in Management of ITP	Completed	Immune Thrombocytopenia	Drug: Caffeic acid tablets Drug: Dexamethasone Drug: Placebo	Study Type: Interventional Phase: Phase 4

## Data Availability

Not applicable.

## References

[1] Cooper N., Bussel J. (2006). The pathogenesis of immune thrombocytopaenic purpura. Br. J. Haematol..

[2] Provan D., Stasi R., Newland A.C. (2010). International consensus report on the investigation and management of primary immune thrombocytopenia. Blood.

[3] Zhang F., Chu X., Wang L., Zhu Y., Li L., Ma D., Peng J., Hou M. (2006). Cell-mediated lysis of autologous platelets in chronic idiopathic thrombocytopenic purpura. Eur. J. Haematol..

[4] Caserta S., Zaccuri A.M., Innao V., Musolino C., Allegra A. (2021). Immune thrombocytopenia: Options and new perspectives. Blood Coagul. Fibrinolysis.

[5] Perera M., Garrido T. (2016). Advances in the pathophysiology of primary immune thrombocytopenia. Hematology.

[6] Kim D.S. (2022). Recent advances in treatments of adult immune thrombocytopenia. Blood Res..

[7] Al-Samkari H., Rosovsky R.P., Leaf R.S.K., Smith D.B., Goodarzi K., Fogerty A.E., Sykes D.B., Kuter D.J. (2020). A modern reassessment of glycoprotein-specific direct platelet autoantibody testing in immune thrombocytopenia. Blood Adv..

[8] Cooper N., Ghanima W. (2019). Immune thrombocytopenia. N. Engl. J. Med..

[9] Hou M., Stockelberg D., Kutti J., Wadenvik H. (1995). Antibodies against platelet GPIb/IX, GPIIb/IIIa, and other platelet antigens in chronic idiopathic thrombocytopenic purpura. Eur. J. Haematol..

[10] McMillan R. (2009). Antiplatelet antibodies in chronic immune thrombocytopenia and their role in platelet destruction and defective platelet production. Hematol. Oncol. Clin. N. Am..

[11] Yu L., Zhang C., Zhang L., Shi Y., Ji X. (2015). Biomarkers for immune thrombocytopenia. Biomark. Res..

[12] Porcelijn L., Schmidt D.E., Oldert G., Egmond S.H.-V., Kapur R., Zwaginga J.J., de Haas M. (2020). Evolution and Utility of Antiplatelet Autoantibody Testing in Patients with Immune Thrombocytopenia. Transfus. Med. Rev..

[13] Wu Z., Zhou J., Prsoon P., Wei X., Liu X., Peng B. (2012). Low expression of FCGRIIB in macrophages of immune thrombocytopenia-affected individuals. Int. J. Hematol..

[14] Moulis G., Germain J., Comont T., Brun N., Dingremont C., Castel B., Arista S., Sailler L., Lapeyre-Mestre M., Beyne-Rauzy O. (2017). Newly diagnosed immune thrombocytopenia adults: Clinical epidemiology, exposure to treatments, and evolution. Results of the CARMEN multicenter prospective cohort. Am. J. Hematol..

[15] Mahévas M., Ebbo M., Audia S., Bonnotte B., Schleinitz N., Durand J., Chiche L., Khellaf M., Bierling P., Roudot-Thoraval F. (2013). Efficacy and safety of rituximab given at 1000 mg on days 1 and 15 compared to the standard regimen to treat adult immune thrombocytopenia. Am. J. Hematol..

[16] Wang Y.-M., Yu Y.-F., Liu Y., Liu S., Hou M., Liu X.-G. (2020). The association between antinuclear antibody and response to rituximab treatment in adult patients with primary immune thrombocytopenia. Hematology.

[17] Baysal M., Baş V., Ümit E., Kırkızlar H.O., Demir A.M. (2022). Could Antinuclear Antibody Positivity Be a Factor Affecting Treatment Response in Immune Thrombocytopenia Patients on Eltrombopag?. Turk. J. Haematol..

[18] Bernlochner I., Goedel A., Plischke C., Schüpke S., Haller B., Schulz C., Mayer K., Morath T., Braun S., Schunkert H. (2015). Impact of immature platelets on platelet response to ticagrelor and prasugrel in patients with acute coronary syndrome. Eur. Heart J..

[19] Benlachgar N., Doghmi K., Masrar A., Mahtat E.M., Harmouche H., Mezalek Z.T. (2020). Immature platelets: A review of the available evidence. Thromb. Res..

[20] Jeon K., Kim M., Lee J., Lee J.-S., Kim H.-S., Kang H.J., Lee Y.K. (2010). Immature platelet fraction: A useful marker for identifying the cause of thrombocytopenia and predicting platelet recovery. Medicine.

[21] Ruisi M.M., Psaila B., Ward M.J., Villarica G., Bussel J.B. (2010). Stability of measurement of the immature platelet fraction. Am. J. Hematol..

[22] Barsam S.J., Psaila B., Forestier M., Page L.K., Sloane P.A., Geyer J.T., Villarica G.O., Ruisi M.M., Gernsheimer T.B., Beer J.H. (2011). Platelet production and platelet destruction: Assessing mechanisms of treatment effect in immune thrombocytopenia. Blood.

[23] Blandinières A., Arlet J.-B., Gaussem P., Pouchot J., Darnige L. (2020). Usefulness of immature platelet fraction measurement for diagnosis and monitoring of iron deficiency associated thrombocytopenia: About two cases. Ann. Biol. Clin..

[24] Negro F., Verdoia M., Tonon F., Nardin M., Kedhi E., De Luca G., the Novara Atherosclerosis Study Group (NAS) (2020). Impact of gender on immature platelet count and its relationship with coronary artery disease. J. Thromb. Thrombolysis.

[25] Verdoia M., Nardin M., Negro F., Tonon F., Gioscia R., Rolla R., De Luca G. (2020). Impact of aging on immature platelet count and its relationship with coronary artery disease. Platelets.

[26] Hong H., Xiao W., Maitta R.W. (2014). Steady Increment of Immature Platelet Fraction Is Suppressed by Irradiation in Single-Donor Platelet Components during Storage. PLoS ONE.

[27] Abe Y., Wada H., Tomatsu H., Sakaguchi A., Nishioka J., Yabu Y., Onishi K., Nakatani K., Morishita Y., Oguni S. (2006). A simple technique to determine thrombopoiesis level using immature platelet fraction (IPF). Thromb. Res..

[28] Frelinger A.L., Grace F., Gerrits A.J., Berny-Lang M., Brown T., Carmichael S.L., Neufeld E., Michelson A.D. (2015). Platelet function tests, independent of platelet count, are associated with bleeding severity in ITP. Blood.

[29] Kurata Y., Hayashi S., Kiyoi T., Kosugi S., Kashiwagi H., Honda S., Tomiyama Y. (2001). Diagnostic Value of Tests for Reticulated Platelets, Plasma Glycocalicin, and Thrombopoietin Levels for Discriminating Between Hyperdestructive and Hypoplastic Thrombocytopenia. Am. J. Clin. Pathol..

[30] Ferreira F.L.B., Colella M.P., Medina S.S., Costa-Lima C., Fiusa M.M.L., Costa L.N.G., Orsi F.A., Annichino-Bizzacchi J.M., Fertrin K.Y., Gilberti M.F.P. (2017). Evaluation of the immature platelet fraction contribute to the differential diagnosis of hereditary, immune and other acquired thrombocytopenias. Sci. Rep..

[31] Adly A.A.M., Ragab I.A., Ismail E.A.R., Farahat M.M. (2015). Evaluation of the immature platelet fraction in the diagnosis and prognosis of childhood immune thrombocytopenia. Platelets.

[32] McDonnell A., Bride K.L., Lim D., Paessler M., Witmer C.M., Lambert M.P. (2018). Utility of the immature platelet fraction in pediatric immune thrombocytopenia: Differentiating from bone marrow failure and predicting bleeding risk. Pediatr. Blood Cancer.

[33] Frelinger A., Grace R.F., Gerrits A.J., Carmichael S.L., Forde E.E., Michelson A.D. (2018). Platelet Function in ITP, Independent of Platelet Count, Is Consistent Over Time and Is Associated with Both Current and Subsequent Bleeding Severity. Thromb. Haemost..

[34] Reeves H.M., Maitta R.W. (2020). Immature Platelet Dynamics in Immune-Mediated Thrombocytopenic States. Front. Med..

[35] Pereira K.N., de Carvalho J.A.M., Paniz C., Moresco R.N., da Silva J.E.P. (2021). Diagnostic characteristics of immature platelet fraction for the assessment of immune thrombocytopenia. Thromb. Res..

[36] Ali I., Graham C., Dempsey-Hibbert N.C. (2019). Immature platelet fraction as a useful marker in the etiological determination of thrombocytopenia. Exp. Hematol..

[37] Serrando M., Marull A., Ruiz M., Del Campo D.P., Puig-Pey I., Muñoz J.M., Tejerina P., Morales-Indiano C. (2016). Clinical significance of IPF% measurement in diagnosing thrombocytopenic disorders: Distinguishing primary immune thrombocytopenia from other disorders. Int. J. Lab. Hematol..

[38] Yang L., Wang L., Zhao C.-H., Zhu X.-J., Hou Y., Jun P., Hou M. (2010). Contributions of TRAIL-mediated megakaryocyte apoptosis to impaired megakaryocyte and platelet production in immune thrombocytopenia. Blood.

[39] Gu D., Chen Z., Zhao H., Du W., Xue F., Ge J., Sui T., Wu H., Liu B., Lu S. (2010). Th1 (CXCL10) and Th2 (CCL2) chemokine expression in patients with immune thrombocytopenia. Hum. Immunol..

[40] Ku F.-C., Tsai C.-R., Der Wang J., Wang C.H., Chang T.-K., Hwang W.-L. (2013). Stromal-derived factor-1 gene variations in pediatric patients with primary immune thrombocytopenia. Eur. J. Haematol..

[41] Wang J.F., Liu Z.Y., Groopman J.E. (1998). The alpha-chemokine receptor CXCR4 is expressed on the megakaryocytic lineage from progenitor to platelets and modulates migration and adhesion. Blood.

[42] Saeidi S., Mohammadi-Asl J., Far M.A.J., Asnafi A.A., Dehuri F., Tavakolifar Y., Saki N. (2017). Is There a Relationship Between CXCR4 Gene Expression and Prognosis of Immune Thrombocytopenia in Children?. Indian J. Hematol. Blood Transfus..

[43] Chevalier N., Jarrossay D., Ho E., Avery D.T., Ma C.S., Yu D., Sallusto F., Tangye S.G., Mackay C.R. (2011). CXCR5 Expressing Human Central Memory CD4 T Cells and Their Relevance for Humoral Immune Responses. J. Immunol..

[44] Ansel K., Harris R.B., Cyster J.G. (2002). CXCL13 Is Required for B1 Cell Homing, Natural Antibody Production, and Body Cavity Immunity. Immunity.

[45] Alvarez E., Piccio L., Mikesell R.J., Klawiter E.C., Parks B.J., Naismith R.T., Cross A.H. (2013). CXCL13 is a biomarker of inflammation in multiple sclerosis, neuromyelitis optica, and other neurological conditions. Mult. Scler. J..

[46] Jernås M., Nookaew I., Wadenvik H., Olsson B. (2013). MicroRNA regulate immunological pathways in T-cells in immune thrombocytopenia (ITP). Blood.

[47] Li J.-Q., Hu S.-Y., Wang Z.-Y., Lin J., Jian S., Dong Y.-C., Wu X.-F., Lan D., Cao L.-J. (2015). MicroRNA-125-5p targeted CXCL13: A potential biomarker associated with immune thrombocytopenia. Am. J. Transl. Res..

[48] Zhang L., Zhou G.-Z., Feng W.-Y., Li D. (2022). Immune Status and Chemokine C Receptor 7 Expression in Primary in Patients with Immune Thrombocytopenia. Turk. J. Hematol..

[49] Schneider P., Mackay F., Steiner V., Hofmann K., Bodmer J.-L., Holler N., Ambrose C., Lawton P., Bixler S., Acha-Orbea H. (1999). BAFF, a Novel Ligand of the Tumor Necrosis Factor Family, Stimulates B Cell Growth. J. Exp. Med..

[50] Moore P.A., Belvedere O., Orr A., Pieri K., LaFleur D.W., Feng P., Soppet D., Charters M., Gentz R., Parmelee D. (1999). BLyS: Member of the Tumor Necrosis Factor Family and B Lymphocyte Stimulator. Science.

[51] Huard B., Schneider P., Mauri D., Tschopp J., French L.E. (2001). T Cell Costimulation by the TNF Ligand BAFF. J. Immunol..

[52] Ye Q., Wang L., Wells A.D., Tao R., Han R., Davidson A., Scott M.L., Hancock W.W. (2004). BAFF binding to T cell-expressed BAFF-R costimulates T cell proliferation and alloresponses. Eur. J. Immunol..

[53] Huard B., Arlettaz L., Ambrose C., Kindler V., Mauri D., Roosnek E., Tschopp J., Schneider P., French L.E. (2004). BAFF production by antigen-presenting cells provides T cell co-stimulation. Int. Immunol..

[54] Zhu X.-J., Shi Y., Peng J., Guo C.-S., Shan N.-N., Qin P., Ji X.-B., Hou M. (2009). The effects of BAFF and BAFF-R-Fc fusion protein in immune thrombocytopenia. Blood.

[55] Khalifa K.A., El-Hawy M.A., Zeid H.M.A., El-Kholy R.M. (2023). Expression of B-cell activating factor in pediatric patients with immune thrombocytopenia: A single institutional series and review of literature. J. Immunoass. Immunochem..

[56] Wu B., Wang W., Zhan Y., Li F., Zou S., Sun L., Cheng Y. (2015). CXCL13, CCL4, and sTNFR as circulating inflammatory cytokine markers in primary and SLE-related autoimmune hemolytic anemia. J. Transl. Med..

[57] Velo-García A., Castro S.G., Isenberg D.A. (2016). The diagnosis and management of the haematologic manifestations of lupus. J. Autoimmun..

[58] Cines D.B., Bussel J.B., Liebman H.A., Prak E.T.L. (2009). The ITP syndrome: Pathogenic and clinical diversity. Blood.

[59] Aringer M., Costenbader K., Daikh D., Brinks R., Mosca M., Ramsey-Goldman R., Smolen J.S., Wofsy D., Boumpas D.T., Kamen D.L. (2019). 2019 European League Against Rheumatism/American College of Rheumatology Classification Criteria for Systemic Lupus Erythematosus. Arthritis Rheumatol..

[60] Dinarello C.A. (2011). Interleukin-1 in the pathogenesis and treatment of inflammatory diseases. Blood.

[61] Calvani N., Tucci M., Richards H.B., Tartaglia P., Silvestris F. (2005). Th1 cytokines in the pathogenesis of lupus nephritis: The role of IL-18. Autoimmun. Rev..

[62] Liang N., Ma W., Yao C., Liu H., Chen X. (2006). Imbalance of interleukin 18 and interleukin 18 binding protein in patients with lupus nephritis. Cell. Mol. Immunol..

[63] Tucci M., Quatraro C., Lombardi L., Pellegrino C., Dammacco F., Silvestris F. (2008). Glomerular accumulation of plasmacytoid dendritic cells in active lupus nephritis: Role of interleukin-18. Arthritis Rheum..

[64] Shan N.-N., Zhu X.-J., Peng J., Qin P., Zhuang X.-W., Wang H.-C., Hou M. (2009). Interleukin 18 and interleukin 18 binding protein in patients with idiopathic thrombocytopenic purpura. Br. J. Haematol..

[65] Sutton C., Brereton C., Keogh B., Mills K.H., Lavelle E.C. (2006). A crucial role for interleukin (IL)-1 in the induction of IL-17–producing T cells that mediate autoimmune encephalomyelitis. J. Exp. Med..

[66] Acosta-Rodriguez E.V., Napolitani G., Lanzavecchia A., Sallusto F. (2007). Interleukins 1beta and 6 but not transforming growth factor-beta are essential for the differentiation of interleukin 17-producing human T helper cells. Nat. Immunol..

[67] Zhan Y., Cheng L., Wu B., Ji L., Chen P., Li F., Cao J., Ke Y., Yuan L., Min Z. (2021). Interleukin (IL)-1 family cytokines could differentiate primary immune thrombocytopenia from systemic lupus erythematosus-associated thrombocytopenia. Ann. Transl. Med..

[68] Sarrand J., Soyfoo M. (2022). Involvement of IL-33 in the Pathophysiology of Systemic Lupus Erythematosus: Review. Int. J. Mol. Sci..

[69] Andersson J. (2007). Cytokines in idiopathic thrombocytopenic purpura (ITP). Acta Paediatr..

[70] Esteller M. (2011). Non-coding RNAs in human disease. Nat. Rev. Genet..

[71] Allegra A., Cicero N., Tonacci A., Musolino C., Gangemi S. (2022). Circular RNA as a Novel Biomarker for Diagnosis and Prognosis and Potential Therapeutic Targets in Multiple Myeloma. Cancers.

[72] Allegra A., Alonci A., Campo S., Penna G., Petrungaro A., Gerace D., Musolino C. (2012). Circulating microRNAs: New biomarkers in diagnosis, prognosis and treatment of cancer (Review). Int. J. Oncol..

[73] Warth S.C., Hoefig K.P., Hiekel A., Schallenberg S., Jovanovic K., Klein L., Kretschmer K., Ansel K.M., Heissmeyer V. (2015). Induced miR-99a expression represses Mtor cooperatively with miR-150 to promote regulatory T-cell differentiation. EMBO J..

[74] Zhao H., Li H., Du W., Zhang D., Ge J., Xue F., Zhou Z., Yang R. (2014). Reduced MIR130A is involved in primary immune thrombocytopenia via targeting TGFB1 and IL18. Br. J. Haematol..

[75] Chang M., Nakagawa P.A., Williams S.A., Schwartz M.R., Imfeld K.L., Buzby J., Nugent D.J. (2003). Immune thrombocytopenic purpura (ITP) plasma and purified ITP monoclonal autoantibodies inhibit megakaryocytopoiesis in vitro. Blood.

[76] Wang J., Peng H., Tian J., Ma J., Tang X., Rui K., Tian X., Wang Y., Chen J.-G., Lu L. (2016). Upregulation of long noncoding RNA TMEVPG1 enhances T helper type 1 cell response in patients with Sjögren syndrome. Immunol. Res..

[77] Liu W.-J., Bai J., Guo Q.-L., Huang Z., Yang H., Bai Y.-Q. (2016). Role of platelet function and platelet membrane glycoproteins in children with primary immune thrombocytopenia. Mol. Med. Rep..

[78] Zhao Y., Cui S., Wang Y., Xu R. (2022). The Extensive Regulation of MicroRNA in Immune Thrombocytopenia. Clin. Appl. Thromb..

[79] Fan Z., Wang X., Li P., Mei C., Zhang M., Zhao C., Song Y. (2020). Systematic Identification of lncRNA-Associated ceRNA Networks in Immune Thrombocytopenia. Comput. Math. Methods Med..

[80] Tremaroli V., Bäckhed F. (2012). Functional interactions between the gut microbiota and host metabolism. Nature.

[81] Allegra A., Innao V., Ettari R., Pugliese M., Pulvirenti N., Musolino C. (2019). Role of the microbiota in hematologic malignancies. Neth. J. Med..

[82] Allegra A., Musolino C., Tonacci A., Pioggia G., Gangemi S. (2020). Interactions between the MicroRNAs and Microbiota in Cancer Development: Roles and Therapeutic Opportunities. Cancers.

[83] Innao V., Allegra A., Musolino C., Allegra A. (2020). New Frontiers about the Role of Human Microbiota in Immunotherapy: The Immune Checkpoint Inhibitors and CAR T-Cell Therapy Era. Int. J. Mol. Sci..

[84] Russell J.T., Roesch L.F.W., Ördberg M., Ilonen J., Atkinson M.A., Schatz D.A., Triplett E.W., Ludvigsson J. (2019). Genetic risk for autoimmunity is associated with distinct changes in the human gut microbiome. Nat. Commun..

[85] Kouchaki E., Tamtaji O.R., Salami M., Bahmani F., Kakhaki R.D., Akbari E., Tajabadi-Ebrahimi M., Jafari P., Asemi Z. (2017). Clinical and metabolic response to probiotic supplementation in patients with multiple sclerosis: A randomized, double-blind, placebo-controlled trial. Clin. Nutr..

[86] Maeda Y., Takeda K. (2017). Role of Gut Microbiota in Rheumatoid Arthritis. J. Clin. Med..

[87] Zhang X., Gu S., You L., Xu Y., Zhou D., Chen Y., Yan R., Jiang H., Li Y., Lv L. (2020). Gut Microbiome and Metabolome Were Altered and Strongly Associated With Platelet Count in Adult Patients With Primary Immune Thrombocytopenia. Front. Microbiol..

[88] Johnsen J. (2012). Pathogenesis in immune thrombocytopenia: New insights. Hematol. Am. Soc. Hematol. Educ. Program.

[89] Lewis S.A., Cines D.B., Cuker A., Semple J.W. (2014). Pathogenesis of immune thrombocytopenia. Presse Med..

[90] Toltl L., Nazi I., Jafari R., Arnold D. (2011). Piecing Together the Humoral and Cellular Mechanisms of Immune Thrombocytopenia. Semin. Thromb. Hemost..

[91] Panzer S., Szamait S., Bödeker R.-H., Haas O.A., Haubenstock A., Mueller-Eckhardt C. (1986). Platelet-associated immunoglobulins IgG, IgM, IgA and complement C3 in immune and nonimmune thrombocytopenic disorders. Am. J. Hematol..

[92] Peerschke E.I., Yin W., Ghebrehiwet B. (2010). Complement activation on platelets: Implications for vascular inflammation and thrombosis. Mol. Immunol..

[93] Kayser W., Mueller-Eckhardt C., Bhakdi S., Ebert K. (1983). Platelet-associated complement C3 in thrombocytopenic states. Br. J. Haematol..

[94] Najaoui A., Bakchoul T., Stoy J., Bein G., Rummel M.J., Santoso S., Sachs U.J. (2012). Autoantibody-mediated complement activation on platelets is a common finding in patients with immune thrombocytopenic purpura (ITP). Eur. J. Haematol..

[95] Peerschke E.I., Andemariam B., Yin W., Bussel J.B. (2010). Complement activation on platelets correlates with a decrease in circulating immature platelets in patients with immune thrombocytopenic purpura. Br. J. Haematol..

[96] Langer H.F., Verschoor A. (2013). Crosstalk between platelets and the complement system in immune protection and disease. Thromb. Haemost..

[97] Lurhuma A.Z., Riccomi H., Masson P.L. (1977). The occurrence of circulating immune complexes and viral antigens in idiopathic thrombocytopenic purpura. Clin. Exp. Immunol..

[98] Trent R.J., Clancy R.L., Danis V., Basten A. (1980). Immune Complexes in Thrombocytopenic Patients: Cause or Effect?. Br. J. Haematol..

[99] Ohali M., Maizlish Y., Abramov H., Schlesinger M., Bransky D., Lugassy G. (2005). Complement profile in childhood immune thrombocytopenic purpura: A prospective pilot study. Ann. Hematol..

[100] Forster J., Katzikadamos Z., Zinn P. (1989). Platelet-associated IgG, IgM, and C3 in paediatric infectious disease. Helv. Paediatr. Acta.

[101] Ruiz-Delgado G.J., Garza E.V., Méndez-Ramírez N., Gómez-Almaguer D. (2009). Abnormalities in the expression of CD55 and CD59 surface molecules on peripheral blood cells are not specific to paroxysmal nocturnal hemoglobinuria. Hematology.

[102] Zhu X., Zhang J., Wang Q., Fu H., Chang Y., Kong Y., Lv M., Xu L., Liu K., Huang X. (2017). Diminished expression of β2-GPI is associated with a reduced ability to mitigate complement activation in anti-GPIIb/IIIa-mediated immune thrombocytopenia. Ann. Hematol..

[103] Sahip B., Pamuk G.E., Uyanik M.S., Pamuk O.N. (2016). Higher interleukin 21 level is predictive of relapse in immune thrombocytopenia. Is it associated with activation of the complement system?. Br. J. Haematol..

[104] Xu C., Zhang R., Duan M., Zhou Y., Bao J., Lu H., Wang J., Hu M., Hu Z., Zhou F. (2022). A polygenic stacking classifier revealed the complicated platelet transcriptomic landscape of adult immune thrombocytopenia. Mol. Ther. Nucleic Acids.

[105] Miltiadous O., Hou M., Bussel J.B. (2020). Identifying and treating refractory ITP: Difficulty in diagnosis and role of combination treatment. Blood.

[106] Psaila B., Bussel J.B. (2008). Refractory immune thrombocytopenic purpura: Current strategies for investigation and management. Br. J. Haematol..

[107] Provan D., Arnold D.M., Bussel J.B., Chong B.H., Cooper N., Gernsheimer T., Ghanima W., Godeau B., González-López T.J., Grainger J. (2019). Updated international consensus report on the investigation and management of primary immune thrombocytopenia. Blood Adv..

[108] Rodeghiero F., Stasi R., Gernsheimer T., Michel M., Provan D., Arnold D.M., Bussel J.B., Cines D.B., Chong B.H., Cooper N. (2009). Standardization of terminology, definitions and outcome criteria in immune thrombocytopenic purpura of adults and children: Report from an international working group. Blood.

[109] Neunert C., Terrell D.R., Arnold D.M., Buchanan G., Cines D.B., Cooper N., Cuker A., Despotovic J.M., George J.N., Grace R.F. (2019). American Society of Hematology 2019 guidelines for immune thrombocytopenia. Blood Adv..

[110] Kim D.J., Chung J.H. (2014). Long-term results of laparoscopic splenectomy in pediatric chronic immune thrombocytopenic purpura. Ann. Surg. Treat. Res..

[111] Cuker A., Neunert C.E. (2016). How I treat refractory immune thrombocytopenia. Blood.

[112] Heitink-Pollé K.M.J., Nijsten J., Boonacker C.W.B., De Haas M., Bruin M.C.A. (2014). Clinical and laboratory predictors of chronic immune thrombocytopenia in children: A systematic review and meta-analysis. Blood.

[113] Bergmann A.K., Grace R.F., Neufeld E.J. (2010). Genetic studies in pediatric ITP: Outlook, feasibility, and requirements. Ann. Hematol..

[114] Rischewski J.R., Imbach P., Paulussen M., Kühne T. (2006). Idiopathic thrombocytopenic purpura (ITP): Is there a genetic predisposition?. Pediatr. Blood Cancer.

[115] Zhao S., Ma J., Zhu X., Zhang J., Wu R. (2021). Chronic Refractory Immune Thrombocytopenia Is Associated With Variants in Immune Genes. Clin. Appl. Thromb..

[116] Strowig T., Henao-Mejia J., Elinav E., Flavell R. (2012). Inflammasomes in health and disease. Nature.

[117] Qiao J., Liu Y., Li X., Xia Y., Wu Y., Li D., Li H., Ma P., Zhu F., Li Z. (2016). Elevated expression of NLRP3 in patients with immune thrombocytopenia. Immunol. Res..

[118] Yu J., Hua M., Zhao X., Wang R., Zhong C., Zhang C., Wang R., Li G., He N., Hou M. (2018). NF-*κ*B-94ins/del ATTG Genotype Contributes to the Susceptibility and Imbalanced Th17 Cells in Patients with Immune Thrombocytopenia. J. Immunol. Res..

[119] Van Niel G., D’Angelo G., Raposo G. (2018). Shedding light on the cell biology of extracellular vesicles. Nat. Rev. Mol. Cell Biol..

[120] Allegra A., Di Gioacchino M., Tonacci A., Petrarca C., Musolino C., Gangemi S. (2021). Multiple Myeloma Cell-Derived Exosomes: Implications on Tumorigenesis, Diagnosis, Prognosis and Therapeutic Strategies. Cells.

[121] Flaumenhaft R., Dilks J.R., Richardson J., Alden E., Patel-Hett S.R., Battinelli E., Klement G.L., Sola-Visner M., Italiano J.E. (2009). Megakaryocyte-derived microparticles: Direct visualization and distinction from platelet-derived microparticles. Blood.

[122] Sewify E.M., Sayed D., Aal R.F.A., Ahmad H.M., Abdou M.A. (2013). Increased circulating red cell microparticles (RMP) and platelet microparticles (PMP) in immune thrombocytopenic purpura. Thromb. Res..

[123] Tantawy A.A.G., Matter R.M., Hamed A.A., Shams El Din El Telbany M.A. (2010). Platelet microparticles in immune thrombocytopenic purpura in pediatrics. Pediatr. Hematol. Oncol..

[124] Álvarez-Román M.T., Fernández-Bello I., Jiménez-Yuste V., Martín-Salces M., Arias-Salgado E.G., Pollmar M.I.R., Sanz R.J., Butta N.V. (2016). Procoagulant profile in patients with immune thrombocytopenia. Br. J. Haematol..

[125] Kao C.-Y., Papoutsakis E.T. (2018). Engineering human megakaryocytic microparticles for targeted delivery of nucleic acids to hematopoietic stem and progenitor cells. Sci. Adv..

[126] Escobar C., Kao C.-Y., Das S., Papoutsakis E.T. (2020). Human megakaryocytic microparticles induce de novo platelet biogenesis in a wild-type murine model. Blood Adv..

[127] Houwerzijl E.J., Blom N.R., van der Want J., Esselink M.T., Koornstra J.J., Smit J.W., Louwes H., Vellenga E., De Wolf J.T.M. (2004). Ultrastructural study shows morphologic features of apoptosis and para-apoptosis in megakaryocytes from patients with idiopathic thrombocytopenic purpura. Blood.

[128] Wang W., Zuo B., Wang Y., Li X., Weng Z., Zhai J., Wu Q., He Y. (2022). Megakaryocyte- and Platelet-Derived Microparticles as Novel Diagnostic and Prognostic Biomarkers for Immune Thrombocytopenia. J. Clin. Med..

[129] Aubert G., Lansdorp P.M. (2008). Telomeres and aging. Physiol. Rev..

[130] Palm W., de Lange T. (2008). How shelterin protects mammalian telomeres. Annu. Rev. Genet..

[131] Epel E.S., Blackburn E.H., Lin J., Dhabhar F.S., Adler N.E., Morrow J.D., Cawthon R.M. (2004). Accelerated telomere shortening in response to life stress. Proc. Natl. Acad. Sci. USA.

[132] Njajou O.T., Cawthon R.M., Damcott C.M., Wu S.-H., Ott S., Garant M.J., Blackburn E.H., Mitchell B.D., Shuldiner A.R., Hsueh W.-C. (2007). Telomere length is paternally inherited and is associated with parental lifespan. Proc. Natl. Acad. Sci. USA.

[133] Kimura M., Cherkas L.F., Kato B.S., Demissie S., Hjelmborg J.B., Brimacombe M., Cupples A., Hunkin J.L., Gardner J.P., Lu X. (2008). Offspring’s leukocyte telomere length, paternal age, and telomere elongation in sperm. PLoS Genet..

[134] Allegra A., Innao V., Penna G., Gerace D., Allegra A.G., Musolino C. (2017). Telomerase and telomere biology in hematological diseases: A new therapeutic target. Leuk. Res..

[135] Hathcock K.S., Chiang Y.J., Hodes R.J. (2005). In vivo regulation of telomerase activity and telomere length. Immunol. Rev..

[136] Klapper W., Moosig F., Sotnikova A., Qian W., Schröder J.O., Parwaresch R. (2004). Telomerase activity in B and T lymphocytes of patients with systemic lupus erythematosus. Ann. Rheum. Dis..

[137] Qi A., Zhou H., Zhou Z., Huang X., Ma L., Wang H., Yang Y., Zhang D., Li H., Ren R. (2013). Telomerase Activity Increased and Telomere Length Shortened in Peripheral Blood Cells from Patients with Immune Thrombocytopenia. J. Clin. Immunol..

[138] Brown T.M., Horblyuk R.V., Grotzinger K.M., Matzdorff A.C., Pashos C.L. (2012). Patient-reported treatment burden of chronic immune thrombocytopenia therapies. BMC Blood Disord..

[139] Bradbury C.A., Pell J., Hill Q., Bagot C., Cooper N., Ingram J., Breheny K., Kandiyali R., Rayment R., Evans G. (2021). Mycophenolate Mofetil for First-Line Treatment of Immune Thrombocytopenia. N. Engl. J. Med..

[140] Gudbrandsdottir S., Birgens H.S., Frederiksen H., Jensen B.A., Jensen M.K., Kjeldsen L., Klausen T.W., Larsen H., Mourits-Andersen H.T., Nielsen C.H. (2013). Rituximab and dexamethasone vs dexamethasone monotherapy in newly diagnosed patients with primary immune thrombocytopenia. Blood.

[141] Schewitz-Bowers L.P., Lait P.J.P., Copland D.A., Chen P., Wu W., Dhanda A.D., Vistica B.P., Williams E.L., Liu B., Jawad S. (2015). Glucocorticoid-resistant Th17 cells are selectively attenuated by cyclosporine A. Proc. Natl. Acad. Sci. USA.

[142] Ramesh R., Kozhaya L., McKevitt K., Djuretic I.M., Carlson T.J., Quintero M.A., McCauley J.L., Abreu M.T., Unutmaz D., Sundrud M.S. (2014). Pro-inflammatory human Th17 cells selectively express P-glycoprotein and are refractory to glucocorticoids. J. Exp. Med..

[143] Xystrakis E., Kusumakar S., Boswell S., Peek E., Urry Z., Richards D.F., Adikibi T., Pridgeon C., Dallman M., Loke T.-K. (2006). Reversing the defective induction of IL-10-secreting regulatory T cells in glucocorticoid-resistant asthma patients. J. Clin. Investig..

[144] Pehlivan M., Okan V., Sever T., Balci S.O., Yilmaz M., Babacan T. (2011). Investigation of TNF-alpha, TGF-beta 1, IL-10, IL-6, IFN-gamma, MBL, GPIA, and IL1A gene polymorphisms in patients with idiopathic thrombocytopenic purpura. Platelets.

[145] Mokhtar G.M., El-Beblawy N.M., Adly A.A., Elbarbary N.S., Kamal T.M., Hasan E.M. (2016). Cytokine gene polymorphism [tumor necrosis factor-alpha (–308), IL-10 (–1082), IL-6 (–174), IL-17F, 1RaVNTR] in pediatric patients with primary immune thrombocytopenia and response to different treatment modalities. Blood Coagul. Fibrinolysis.

[146] Stimpson M.L., Lait P.J., Schewitz-Bowers L.P., Williams E.L., Thirlwall K.F., Lee R.W., Bradbury C.A. (2020). IL-10 and IL-17 expression by CD4+ T cells is altered in corticosteroid refractory immune thrombocytopenia (ITP). J. Thromb. Haemost..

[147] Stimpson M.L., Wolf J.S., Williams E.L., Lait P.J.P., Schewitz-Bowers L.P., Greenwood R., Pell J., Thomas I., Lee R.W.J., Bradbury C.A. (2022). CD4 ^+^ T cells from patients with glucocorticoid-refractory immune thrombocytopenia have altered cytokine expression. Br. J. Haematol..

[148] Richards D.F., Fernandez M., Caulfield J., Hawrylowicz C.M. (2000). Glucocorticoids drive human CD8(+) T cell differentiation towards a phenotype with high IL-10 and reduced IL-4, IL-5 and IL-13 production. Eur. J. Immunol..

[149] Ma L., Simpson E., Li J., Xuan M., Xu M., Baker L., Shi Y., Yougbaré I., Wang X., Zhu G. (2015). CD8+ T cells are predominantly protective and required for effective steroid therapy in murine models of immune thrombocytopenia. Blood.

[150] Hua F., Ji L., Zhan Y., Li F., Zou S., Wang X., Song D., Min Z., Gao S., Wu Y. (2012). Pulsed High-dose Dexamethasone Improves Interleukin 10 Secretion by CD5+ B Cells in Patients with Primary Immune Thrombocytopenia. J. Clin. Immunol..

[151] Webster M.L., Sayeh E., Crow M., Chen P., Nieswandt B., Freedman J., Ni H. (2006). Relative efficacy of intravenous immunoglobulin G in ameliorating thrombocytopenia induced by antiplatelet GPIIbIIIa versus GPIbalpha antibodies. Blood.

[152] Zeng Q., Zhu L., Tao L., Bao J., Yang M., Simpson E.K., Li C., van der Wal D.E., Chen P., Spring C.M. (2012). Relative efficacy of steroid therapy in immune thrombocytopenia mediated by anti-platelet GPIIbIIIa versus GPIbalpha antibodies. Am. J. Hematol..

[153] Peng J., Ma S., Liu J., Hou Y., Liu X., Niu T., Xu R., Guo C., Wang X., Cheng Y. (2014). Association of autoantibody specificity and response to intravenous immunoglobulin G therapy in immune thrombocytopenia: A multicenter cohort study. J. Thromb. Haemost..

[154] Li J., van der Wal D.E., Zhu G., Xu M., Yougbare I., Ma L., Vadasz B., Carrim N., Grozovsky R., Ruan M. (2015). Desialylation is a mechanism of Fc-independent platelet clearance and a therapeutic target in immune thrombocytopenia. Nat. Commun..

[155] Li J., Callum J.L., Lin Y., Zhou Y., Zhu G., Ni H. (2014). Severe platelet desialylation in a patient with glycoprotein Ib/IX antibody-mediated immune thrombocytopenia and fatal pulmonary hemorrhage. Haematologica.

[156] Tao L., Zeng Q., Li J., Xu M., Wang J., Pan Y., Wang H., Tao Q., Chen Y., Peng J. (2017). Platelet desialylation correlates with efficacy of first-line therapies for immune thrombocytopenia. J. Hematol. Oncol..

[157] Peschke B., Keller C.W., Weber P., Quast I., Lünemann J.D. (2017). Fc-Galactosylation of Human Immunoglobulin Gamma Isotypes Improves C1q Binding and Enhances Complement-Dependent Cytotoxicity. Front. Immunol..

[158] Sonneveld M.E., de Haas M., Koeleman C., de Haan N., Zeerleder S.S., Ligthart P.C., Wuhrer M., van der Schoot C.E., Vidarsson G. (2017). Patients with IgG1-anti-red blood cell autoantibodies show aberrant Fc-glycosylation. Sci. Rep..

[159] Vučković F., Krištić J., Gudelj I., Teruel M., Keser T., Pezer M., Pučić-Baković M., Štambuk J., Trbojević-Akmačić I., Barrios C. (2015). Association of systemic lupus erythematosus with decreased immunosuppressive potential of the IgG glycome. Arthritis Rheumatol..

[160] Šimurina M., de Haan N., Vučković F., Kennedy N.A., Štambuk J., Falck D., Trbojević-Akmačić I., Clerc F., Razdorov G., Khon A. (2018). Glycosylation of Immunoglobulin G Associates With Clinical Features of Inflammatory Bowel Diseases. Gastroenterology.

[161] Su Z., Xie Q., Wang Y., Li Y. (2020). Aberrant Immunoglobulin G Glycosylation in Rheumatoid Arthritis by LTQ-ESI-MS. Int. J. Mol. Sci..

[162] Sjöwall C., Zapf J., Von Löhneysen S., Magorivska I., Biermann M., Janko C., Winkler S., Bilyy R., Schett G., Herrmann M. (2015). Altered glycosylation of complexed native IgG molecules is associated with disease activity of systemic lupus erythematosus. Lupus.

[163] Fokkink W.J., Selman M.H., Dortland J.R., Durmuş B., Kuitwaard K., Huizinga R., van Rijs W., Tio-Gillen A.P., van Doorn P.A., Deelder A.M. (2014). IgG Fc Nglycosylation in Guillain-Barré syndrome treated with immunoglobulins. J. Proteome Res..

[164] Wang W., Xu X., Huang C., Gao C. (2022). N-glycan profiling alterations of serum and immunoglobulin G in immune thrombocytopenia. J. Clin. Lab. Anal..

[165] Bakchoul T., Walek K., Krautwurst A., Rummel M., Bein G., Santoso S., Sachs U.J. (2013). Glycosylation of autoantibodies: Insights into the mechanisms of immune thrombocytopenia. Thromb. Haemost..

[166] Wu D., Struwe W.B., Harvey D.J., Ferguson M.A.J., Robinson C.V. (2018). N-glycan microheterogeneity regulates interactions of plasma proteins. Proc. Natl. Acad. Sci. USA.

[167] Walsh M.C., Lee J., Choi Y. (2015). Tumor necrosis factor receptor-associated factor 6 (TRAF6) regulation of development, function, and homeostasis of the immune system. Immunol. Rev..

[168] Liu H., Tamashiro S., Baritaki S., Penichet M., Yu Y., Chen H., Berenson J., Bonavida B. (2012). TRAF6 Activation in Multiple Myeloma: A Potential Therapeutic Target. Clin. Lymphoma Myeloma Leuk..

[169] Rhyasen G.W., Bolanos L., Fang J., Jerez A., Wunderlich M., Rigolino C., Mathews L., Ferrer M., Southall N., Guha R. (2013). Targeting IRAK1 as a Therapeutic Approach for Myelodysplastic Syndrome. Cancer Cell.

[170] Asoglu V., Umit E.G., Demir M. (2019). A biomarker and therapeutical target in immune thrombocytopenia: TNF receptor-associated factor 6. Biomark. Med..

[171] Kojouri K., Vesely S., Terrell D., George J.N. (2004). Splenectomy for adult patients with idiopathic thrombocytopenic purpura: A systematic review to assess long-term platelet count responses, prediction of response, and surgical complications. Blood.

[172] Fabris F., Tassan T., Ramon R., Carraro G., Randi M.L., Luzzatto G., Moschino P., Girolami A. (2001). Age as the major predictive factor of long-term response to splenectomy in immune thrombocytopenic purpura. Br. J. Haematol..

[173] Katkhouda N., Grant S.W., Mavor E., Friedlander M.H., Lord R.V.N., Achanta K., Essani R., Mason R. (2001). Predictors of response after laparoscopic splenectomy for immune thrombocytopenic purpura. Surg. Endosc..

[174] Kumar S., Diehn F., Gertz M., Tefferi A. (2002). Splenectomy for immune thrombocytopenic purpura: Long-term results and treatment of postsplenectomy relapses. Ann. Hematol..

[175] Vianelli N., Galli M., de Vivo A., Intermesoli T., Giannini B., Mazzucconi M.G., Barbui T., Tura S., Baccaranion M. (2005). Gruppo Italiano per lo Studio delle Malattie Ematologiche dell’Adulto: Efficacy and safety of splenectomy in immune thrombocytopenic purpura: Long-term results of 402 cases. Haematologica.

[176] Bourgeois E., Caulier M.T., Delarozee C., Brouillard M., Bauters F., Fenaux P. (2003). Long-term follow-up of chronic autoimmune thrombocytopenic purpura refractory to splenectomy: A prospective analysis. Br. J. Haematol..

[177] Radaelli F., Faccini P., Goldaniga M., Guggiari E., Pozzoli E., Maiolo A.T., Ciani A., Pogliani E.M. (2000). Factors predicting response to splenectomy in adult patients with idiopathic thrombocytopenic purpura. Haematologica.

[178] Wu J.-M., Lai I.-R., Yuan R.-H., Yu S.-C. (2004). Laparoscopic splenectomy for idiopathic thrombocytopenic purpura. Am. J. Surg..

[179] Balague C., Vela S., Targarona E.M., Gich I.J., Muniz E., D’Ambra A., Pey A., Monllau V., Ascaso E., Martinez C. (2006). Predictive factors for successful laparoscopic splenectomy in immune thrombocytopenic purpura: Study of clinical and laboratory data. Surg. Endosc..

[180] Najean Y., Rain J., Billotey C. (1997). The site of destruction of autologous ^111^ In-labelled platelets and the efficiency of splenectomy in children and adults with idiopathic thrombocytopenic purpura: A study of 578 patients with 268 splenectomies. Br. J. Haematol..

[181] Oliviero S., Morrone G., Cortese R. (1987). The human haptoglobin gene: Transcriptional regulation during development and acute phase induction. EMBO J..

[182] Levy A.P., Asleh R., Blum S., Levy N.S., Miller-Lotan R., Kalet-Litman S., Anbinder Y., Lache O., Nakhoul F.M., Asaf R. (2010). Haptoglobin: Basic and Clinical Aspects. Antioxid. Redox Signal..

[183] Langlois M.R., Delanghe J.R. (1996). Biological and clinical significance of haptoglobin polymorphism in humans. Clin. Chem..

[184] Pavón E.J., Muñoz P., Lario A., Longobardo V., Carrascal M., Abián J., Martín A.B., Arias S.A., Callejas-Rubio J.-L., Sola R. (2006). Proteomic analysis of plasma from patients with systemic lupus erythematosus: Increased presence of haptoglobin α2 polypeptide chains over the α1 isoforms. Proteomics.

[185] Mao L., Dong H., Yang P., Zhou H., Huang X., Lin X., Kijlstra A. (2008). MALDI-TOF/TOF-MS Reveals Elevated Serum Haptoglobin and Amyloid A in Behcet’s Disease. J. Proteome Res..

[186] Zheng C.-X., Ji Z.-Q., Zhang L.-J., Wen Q., Chen L.-H., Yu J.-F., Zheng D. (2012). Proteomics-based identification of haptoglobin as a favourable serum biomarker for predicting long-term response to splenectomy in patients with primary immune thrombocytopenia. J. Transl. Med..

[187] Murotomi K., Umeno A., Shichiri M., Tanito M., Yoshida Y. (2023). Significance of Singlet Oxygen Molecule in Pathologies. Int. J. Mol. Sci..

[188] Malhotra J.D., Kaufman R.J. (2007). Endoplasmic reticulum stress and oxidative stress: A vicious cycle or a double-edged sword?. Antioxid. Redox Signal..

[189] Kamata H., Hirata H. (1999). Redox Regulation of Cellular Signalling. Cell. Signal..

[190] Schafer F.Q., Buettner G.R. (2001). Redox environment of the cell as viewed through the redox state of the glutathione disulfide/glutathione couple. Free Radic. Biol. Med..

[191] Agarwal A., Saleh R.A., Bedaiwy M.A. (2003). Role of reactive oxygen species in the pathophysiology of human reproduction. Fertil. Steril..

[192] Afzal A., Nicosia N., Fumia A., Giorgianni F., Santini A., Cicero N. (2022). Resveratrol and Immune Cells: A Link to Improve Human Health. Molecules.

[193] Ben Bakrim W., Aghraz A., Hriouch F., Larhsini M., Markouk M., Bekkouche K., Costa R., Arrigo S., Cicero N., Dugo G. (2022). Phytochemical study and antioxidant activity of the most used medicinal and aromatic plants in Morocco. J. Essent. Oil Res..

[194] Alesci A., Miller A., Tardugno R., Pergolizzi S. (2022). Chemical analysis, biological and therapeutic activities of *Olea europaea* L. extracts. Nat. Prod. Res..

[195] Allegra A., Pioggia G., Tonacci A., Musolino C., Gangemi S. (2020). Oxidative Stress and Photodynamic Therapy of Skin Cancers: Mechanisms, Challenges and Promising Developments. Antioxidants.

[196] Musolino C., Allegra A., Saija A., Alonci A., Russo S., Spatari G., Penna G., Gerace D., Cristani M., David A. (2012). Changes in advanced oxidation protein products, advanced glycation end products, and s-nitrosylated proteins, in patients affected by polycythemia vera and essential thrombocythemia. Clin. Biochem..

[197] Gangemi S., Allegra A., Alonci A., Cristani M., Russo S., Speciale A., Penna G., Spatari G., Cannavò A., Bellomo G. (2012). Increase of novel biomarkers for oxidative stress in patients with plasma cell disorders and in multiple myeloma patients with bone lesions. Inflamm. Res..

[198] Musolino C., Allegra A., Alonci A., Saija A., Russo S., Cannavò A., Cristani M., Centorrino R., Saitta S., Alibrandi A. (2011). Carbonyl group serum levels are associated with CD38 expression in patients with B chronic lymphocytic leukemia. Clin. Biochem..

[199] Cristani M., Speciale A., Saija A., Gangemi S., Minciullo P., Cimino F. (2016). Circulating Advanced Oxidation Protein Products as Oxidative Stress Biomarkers and Progression Mediators in Pathological Conditions Related to Inflammation and Immune Dysregulation. Curr. Med. Chem..

[200] Kurien B.T., Scofield R.H. (2008). Autoimmunity and oxidatively modified autoantigens. Autoimmun. Rev..

[201] Kurien B.T., Hensley K., Bachmann M., Scofield R.H. (2006). Oxidatively modified autoantigens in autoimmune diseases. Free Radic. Biol. Med..

[202] Zhang B., Zehnder J.L. (2013). Oxidative Stress and Immune Thrombocytopenia. Semin. Hematol..

[203] Amini L., Kaeda J., Fritsche E., Roemhild A., Kaiser D., Reinke P. (2023). Clinical adoptive regulatory T Cell therapy: State of the art, challenges, and prospective. Front. Cell Dev. Biol..

[204] Stasi R., Cooper N., Del Poeta G., Stipa E., Evangelista M.L., Abruzzese E., Amadori S. (2008). Analysis of regulatory T-cell changes in patients with idiopathic thrombocytopenic purpura receiving B cell–depleting therapy with rituximab. Blood.

[205] Olsson B., Ridell B., Carlsson L., Jacobsson S., Wadenvik H. (2008). Recruitment of T cells into bone marrow of ITP patients possibly due to elevated expression of VLA-4 and CX3CR1. Blood.

[206] Semple J.W. (2008). ITP three R’s: Regulation, routing, rituximab. Blood.

[207] Brahmachari S., Pahan K. (2010). Myelin Basic Protein Priming Reduces the Expression of Foxp3 in T Cells via Nitric Oxide. J. Immunol..

[208] Zhang B., Lo C., Shen L., Sood R., Jones C., Cusmano-Ozog K., Park-Snyder S., Wong W., Jeng M., Cowan T. (2011). The role of vanin-1 and oxidative stress–related pathways in distinguishing acute and chronic pediatric ITP. Blood.

[209] Akbayram S., Doğan M., Akgün C., Mukul Y., Peker E., Bay A., Çaksen H., Oner A.F. (2010). The Association of Oxidant Status and Antioxidant Capacity in Children With Acute and Chronic ITP. J. Pediatr. Hematol..

[210] Polat G., Tamer L., Taniriverdi K., Gürkan E., Baslamisli F., Atik U., Gorkan E. (2002). Levels of malondialdehyde, glutathione and ascorbic acid in idiopathic thrombocytopaenic purpura. East Afr. Med. J..

[211] Rasheed Z., Khan M.W.A., Ali R. (2006). Hydroxyl radical modification of human serum albumin generated cross reactive antibodies. Autoimmunity.

[212] Jin C.-Q., Dong H.-X., Cheng P.-P., Zhou J.-W., Zheng B.-Y., Liu F. (2013). Antioxidant Status and Oxidative Stress in Patients with Chronic ITP. Scand. J. Immunol..

[213] Kamhieh-Milz J., Bal G., Sterzer V., Gurkan E., Baslamisli F., Atik U. (2012). Reduced antioxidant capacities in platelets from patients with autoimmune thrombocytopenia purpura (ITP). Platelets.

[214] Lee I.-T., Yang C.-M. (2012). Role of NADPH oxidase/ROS in pro-inflammatory mediators-induced airway and pulmonary diseases. Biochem. Pharmacol..

[215] Iuchi Y., Kibe N., Tsunoda S., Suzuki S., Mikami T., Okada F., Uchida K., Fujii J. (2010). Implication of oxidative stress as a cause of autoimmune hemolytic anemia in NZB mice. Free Radic. Biol. Med..

[216] Yu J., Heck S., Patel V., LeVan J., Yu Y., Bussel J.B., Yazdanbakhsh K. (2008). Defective circulating CD25 regulatory T cells in patients with chronic immune thrombocytopenic purpura. Blood.

[217] Elalfy M.S., Elhenawy Y.I., Deifalla S., Hegazy M., Sabra A., Abdelaziz Y. (2015). Oxidant/antioxidant status in children and adolescents with immune thrombocytopenia (ITP) and the role of an adjuvant antioxidant therapy. Pediatr. Blood Cancer.

[218] Yacobovich J., Revel-Vilk S., Tamary H. (2013). Childhood Immune Thrombocytopenia—Who Will Spontaneously Recover?. Semin. Hematol..

[219] Chen J., Lu H., Wang X., Yang J., Luo J., Wang L., Yi X., He Y., Chen K. (2022). VNN1 contributes to the acute kidney injury-chronic kidney disease transition by promoting cellular senescence via affecting RB1 expression. FASEB J..

[220] Sincan G., Erdem F., Bay I., Sincan S. (2022). Serum Copper and Zinc Levels in Primary Immune Thrombocytopenia. Biol. Trace Element Res..

[221] Chen R., Zou J., Kang R., Tang D. (2023). The redox protein HMGB1 in cell death and cancer. Antioxid. Redox Signal..

[222] Murdaca G., Allegra A., Paladin F., Calapai F., Musolino C., Gangemi S. (2021). Involvement of Alarmins in the Pathogenesis and Progression of Multiple Myeloma. Int. J. Mol. Sci..

[223] Lu M., Yu S., Xu W., Gao B., Xiong S. (2015). HMGB1 Promotes Systemic Lupus Erythematosus by Enhancing Macrophage Inflammatory Response. J. Immunol. Res..

[224] Zhang G., Yang P., Liu X., Liu H., Wang J., Wang J., Xiao J., Nie D., Ma L. (2022). HMGB1 is increased in patients with immune thrombocytopenia and negatively associates with Tregs. Thromb. Res..

[225] Wang R., Cao Q., Bai S.-T., Wang L., Sheng G.-Y. (2019). Potential role and mechanism for high mobility group box1 in childhood chronic immune thrombocytopenia. Eur. Rev. Med. Pharmacol. Sci..

[226] Cura M., Koç A., Aksoy N., Özdemir Z.C. (2016). Effect of short-term, high-dose methylprednisolone on oxidative stress in children with acute immune thrombocytopenia. Blood Res..

[227] Brox A.G., Howson-Jan K., Fauser A.A. (1988). Treatment of idiopathic thrombocytopenic purpura with ascorbate. Br. J. Haematol..

[228] Masugi J., Iwai M., Kimura S., Ochi F., Suzuki K., Nakano O., Sakamoto T., Fukunaga H., Amano M., Fukuda T. (1994). Combination of Ascorbic Acid and Methylprednisolone Pulse Therapy in the Treatment of Idiopathic Thrombocytopenic Purpura. Intern. Med..

[229] Cohen H.A., Nussinovitch M., Gross S., Hart J., Frydman M. (1993). Treatment of Chronic Idiopathic Thrombocytopenic Purpura with Ascorbate. Clin. Pediatr..

[230] Rossetti F., Labate P., Pillon M. (1992). L-Ascorbic Acid for the Treatment of Childhood Chronic Idiopathic Thrombocytopenic Purpura. Pediatr. Hematol. Oncol..

[231] Jubelirer S.J. (1993). Pilot study of ascorbic acid for the treatment of refractory immune thrombocytopenic purpura. Am. J. Hematol..

[232] Amendola G., Cirillo G., Spiezie M., Di Concilio R., Rolando P. (1995). Treatment of Childhood Chronic Idiopathic Thrombocytopenic Purpura With Ascorbate. Clin. Pediatr..

[233] www.ClinicalTrials.gov.

[234] Beyazit H., Demiryürek A.T., Temel M.T., Pekpak E., Demiryürek S., Akbayram S. (2019). Investigation of Dynamic Thiol/Disulfide Homeostasis in Children With Acute Immune Thrombocytopenia. J. Pediatr. Hematol..

[235] Li N., Mahamad S., Parpia S., Iorio A., Foroutan F., Heddle N.M., Hsia C.C., Sholzberg M., Rimmer E., Shivakumar S. (2022). Development and internal validation of a clinical prediction model for the diagnosis of immune thrombocytopenia. J. Thromb. Haemost..

[236] Bay A., Coskun E., Oztuzcu S., Ergun S., Yilmaz F., Aktekin E. (2014). Plasma microRNA profiling of pediatric patients with immune thrombocytopenic purpura. Blood Coagul. Fibrinolysis.

[237] Chen J.-F., Yang L.-H., Feng J.-J., Chang L.-X., Liu X.-E., Lu Y.-J. (2010). The clinical significance of immune-related marker detection in idiopathic thrombocytopenic purpura. Zhonghua Nei Ke Za Zhi.

[238] Tag L.M., Ezz-Eldeen A.M., Mahmoud M.S., Rashed H.-A.G., Noaman H.A. (2004). Serum IL-2 and platelet-associated immunoglobulins are good prognostic markers in immune thrombocytopenic purpura. Egypt. J. Immunol..

[239] Wu L.C., Tuot D.S., Lyons D.S., Garcia K.C., Davis M.M. (2002). Two-step binding mechanism for T-cell receptor recognition of peptide–MHC. Nature.

[240] Davis M.M., Boniface J.J., Reich Z., Lyons D., Hampl J., Arden B., Chien Y. (1998). Ligand recognition by alpha beta T cell receptors. Annu. Rev. Immunol..

[241] Mallajosyula V., Ganjavi C., Chakraborty S., McSween A.M., Pavlovitch-Bedzyk A.J., Wilhelmy J., Nau A., Manohar M., Nadeau K.C., Davis M.M. (2021). CD8 ^+^ T cells specific for conserved coronavirus epitopes correlate with milder disease in patients with COVID-19. Sci. Immunol..

[242] Ou C., Chang H., Hung Y.-S., Kuo M.-C., Li P.-L., Lin T.-L. (2022). Changing profile of platelet activity and turnover indices during treatment response of immune thrombocytopenia. Clin. Exp. Med..

